# Supplementary material for: Spermidine from arginine metabolism activates Nrf2 and inhibits kidney fibrosis
Source: Commun Biol. 2023 Jun 28;6:676. doi: 10.1038/s42003-023-05057-w (PMC10307812; doi:10.1038/s42003-023-05057-w)
Supplement: Supplementary file 2 — Supplementary Information [file 42003_2023_5057_MOESM2_ESM.pdf]

Supplemental Material for

Spermidine from arginine metabolism activates Nrf2 and inhibits kidney fibrosis

Seishi Aihara, MD,[1] Kumiko Torisu, MD, PhD,[1,2,*] Yushi Uchida, MD, PhD,[1] Noriyuki Imazu, MD,[1] Toshiaki Nakano, MD, PhD,[1,3,*] Takanari Kitazono, MD, PhD[1]

[1]Department of Medicine and Clinical Science, Graduate School of Medical Sciences, Kyushu University, Fukuoka, Japan

[2]Department of Integrated Therapy for Chronic Kidney Disease, Graduate School of Medical Sciences, Kyushu University, Fukuoka, Japan

[3]Center for Cohort Studies, Graduate School of Medical Sciences, Kyushu University 3-1-1 Maidashi, Higashi-ku, Fukuoka, Japan

Corresponding authors

Kumiko Torisu, M.D., Ph.D.

Department of Integrated Therapy for Chronic Kidney Disease, Graduate School of Medical Sciences, Kyushu University, 3-1-1 Maidashi, Higashi-ku, Fukuoka 812-8582, Japan.

Tel.: +81-92-642-5843; fax: +81-92-642-5846

E-mail: torisu.kumiko.350@m.kyushu-u.ac.jp

Toshiaki Nakano, MD, PhD

Center for Cohort Studies, Graduate School of Medical Sciences, Kyushu University 3-1-1 Maidashi, Higashi-ku, Fukuoka 812-8582, Japan

Tel: +81-92-642-5843; fax: +81-92-642-5846

E-mail: nakano.toshiaki.455@m.kyushu-u.ac.jp

Supplementary Figures

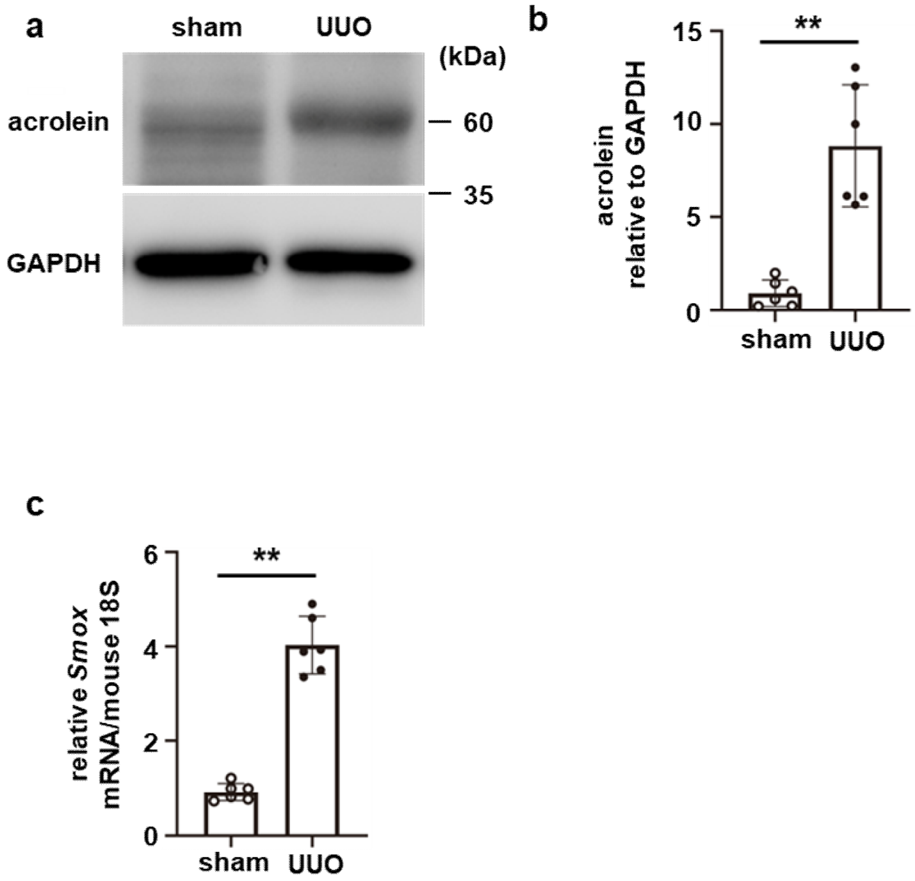

Supplementary Fig. S1. *Smox* mRNA expression and acrolein protein levels are increased in the UUO kidney.

(a) Western blot analysis of acrolein in sham and UUO kidneys. **(b)** Quantification of relative levels of acrolein protein normalized to GAPDH is shown (n = 6 in each group). Data are indicated as means ± SD. **(c)** *Smox* mRNA expression determined by real-time PCR in sham and UUO kidneys (n = 6 in each group). Data are indicated as means ± SD. $^{**}p < 0.01$. UUO, unilateral ureteral obstruction; GAPDH, glyceraldehyde-3-phosphate dehydrogenase; *Smox*, spermine oxidase.

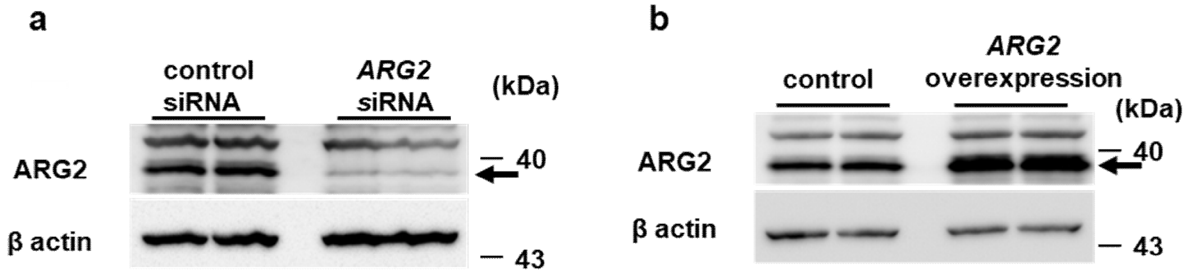

Supplementary Fig. S2. Confirmation of *Arg2* knockdown and overexpression by western blot analysis.

Western blot analysis of ARG2 in HK-2 cells **(a)** transfected with control- or *Arg2*-siRNA, and **(b)** overexpression of ARG2. The arrows indicate ARG2 protein. ARG2, arginase 2.

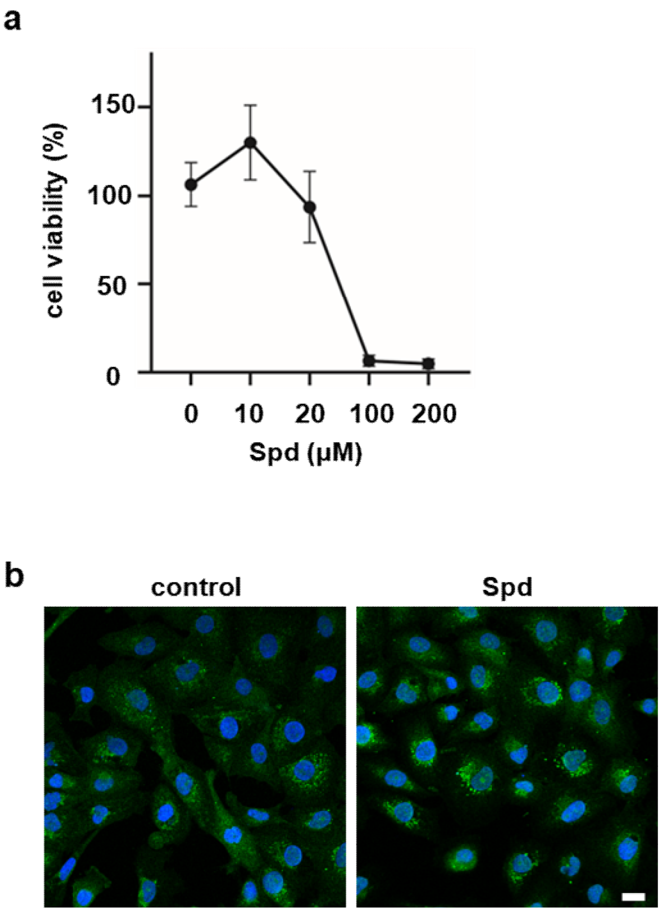

Supplementary Fig. S3. Intracellular spermidine levels increase when spermidine is added to HK-2 cells.

(a) Cell viability of HK-2 cells exposed to graded concentrations of Spd. Data are indicated as means ± SD. **(b)** Spd staining of control or Spd-treated cells. Spd-positive puncta can be seen in the cytoplasm. Scale bar, 20 μm. Spd, spermidine.

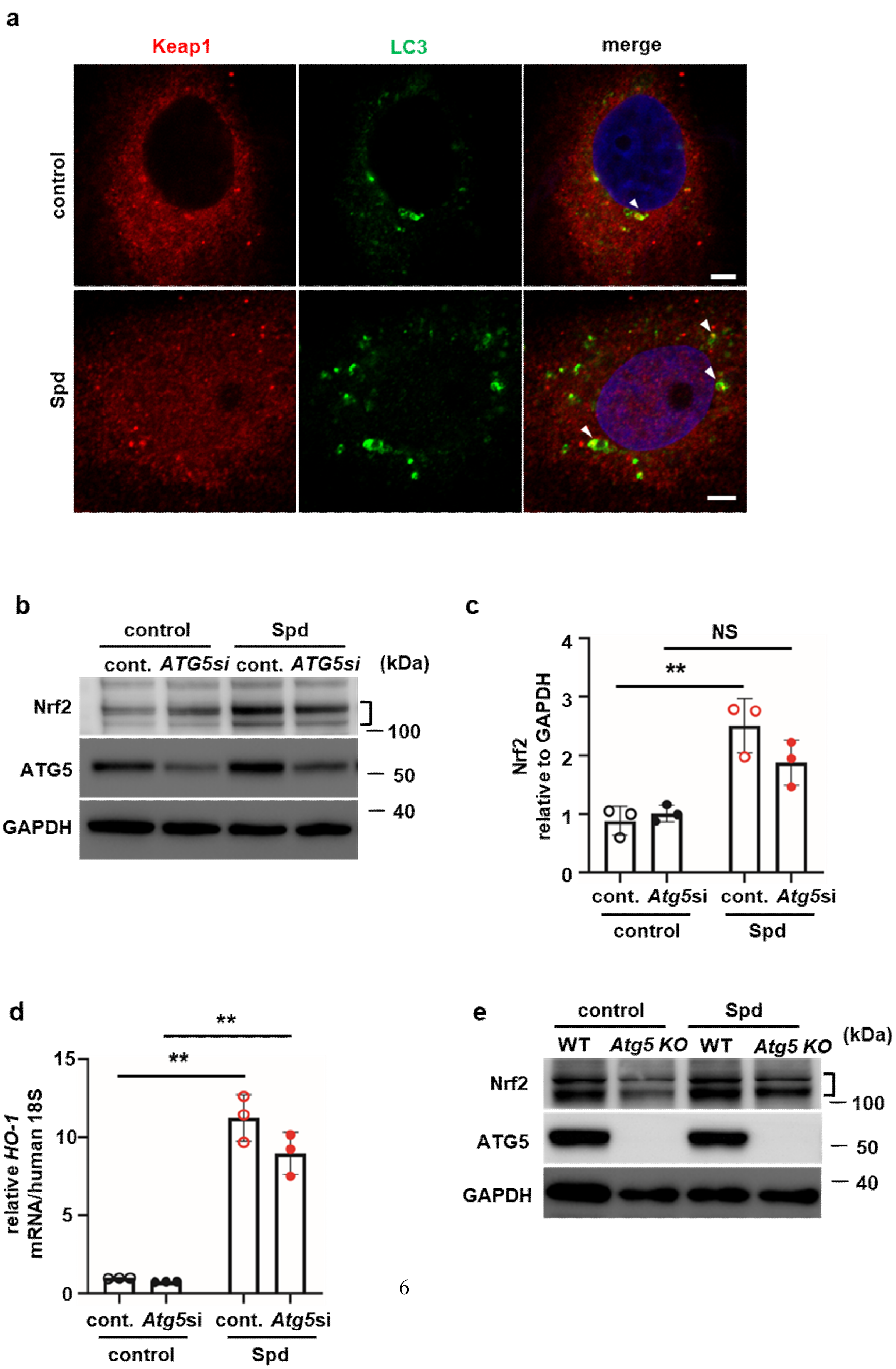
a
Keap1
LC3
merge
control
Spd
b
control
Spd
cont. ATG5si cont. ATG5si
(kDa)
Nrf2
ATG5
GAPDH
100
50
40
c
NS
**
Nrf2
relative to GAPDH
0
1
2
3
4
cont. Atg5si cont. Atg5si
control
Spd
d
**
**
relative HO-1
mRNA/human 18S
0
5
10
15
cont. Atg5si cont. Atg5si
control
Spd
e
control
Spd
WT Atg5 KO WT Atg5 KO
(kDa)
Nrf2
ATG5
GAPDH
100
50
40

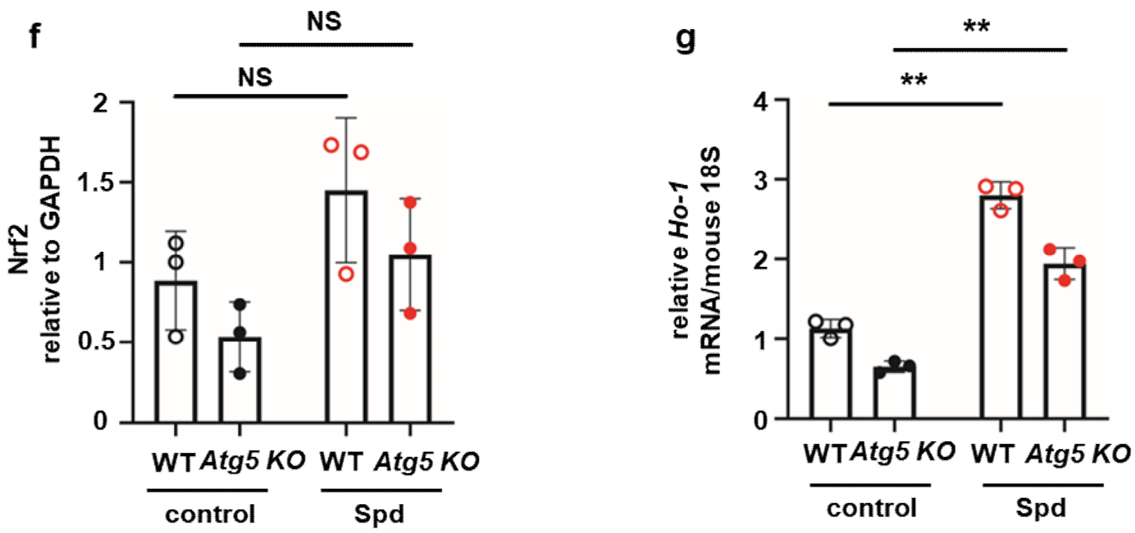

Supplementary Fig. S4. Autophagy is partly involved in the activation of Nrf2 by Spd. **(a)** Confocal immunofluorescence microscopic images of Keap1 and LC3 in control or Spd-treated HK-2 cells. Red, anti-Keap1 antibody; green, anti-LC3 antibody; blue, DAPI. White arrowheads indicate co-localization areas. Scale bars, 5 µm. (**b**) Western blot analysis of Nrf2 and ATG5 in Spd-treated control and *ATG5* knockdown tubular cells. (**c**) Relative levels of Nrf2 protein normalized to GAPDH are shown (n = 3 in each group). (**d**) *HO-1* mRNA expression determined by real-time PCR in control and *ATG5* knockdown HK-2 cells incubated with Spd (n = 3 in each group). Data are indicated as means ± SD. (**e**) Western blot analysis of Nrf2 and ATG5 in Spd-treated WT and *Atg5* KO MEFs. Data are indicated as means ± SD. (**f**) Quantification of Nrf2 protein levels in Spd-treated WT and *Atg5* KO MEFs (n = 3 in each group). Data are indicated as means ± SD. (**g**) *Ho-1* mRNA expression determined by real-time PCR in

Spd-treated WT and *Atg5* KO MEFs (n = 3 in each group). Data are indicated as means ± SD. Spd, spermidine; MEF, mouse embryonic fibroblast; LC3, microtubule-associated protein 1A/1B-light chain 3; Keap1, kelch-like ECH-associated protein 1; DAPI, blue, 4′, 6-diamidino-2-phenylindole; Nrf2, nuclear factor erythroid 2-related factor 2; ATG5, autophagy-related 5.

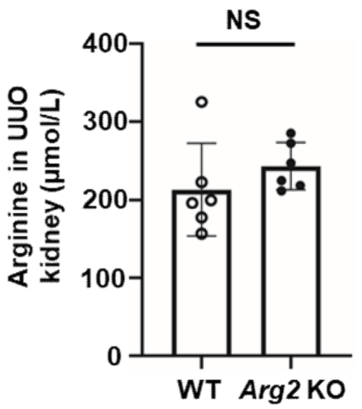

Supplementary Fig. S5. Arginine content in kidneys of WT and *Arg2* KO mice.

L-arginine concentrations in the UUO kidney of WT and *Arg2* KO mice as measured by enzyme-linked immunosorbent assay (ELISA, n = 6 in each group). Data are indicated as means ± SD. WT, wild-type; UUO, unilateral ureteral obstruction; *Arg2*, arginase 2.

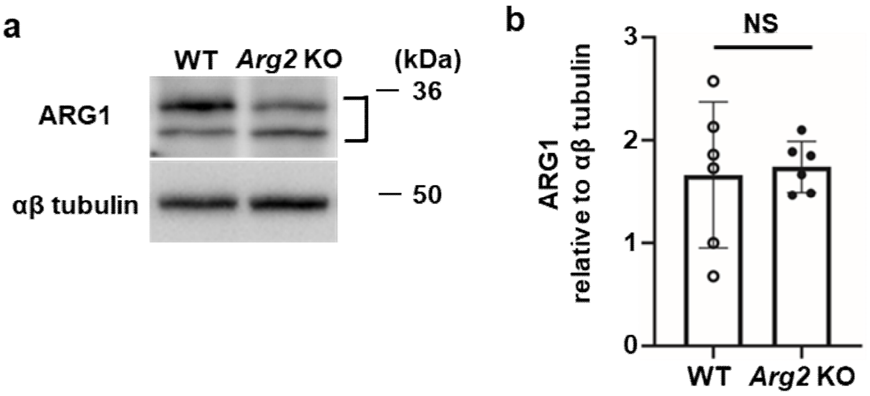

Supplementary Fig. S6. ARG1 is not increased in the *Arg2* KO UUO kidney.

(a) Western blot analysis of ARG1 protein in the UUO kidney of WT and *Arg2* KO mice.

(b) Quantification of relative levels of ARG1 protein normalized to αβ tubulin is shown on the right (n = 6 in each group). Data are indicated as means ± SD. UUO, unilateral ureteral obstruction; ARG1, arginase 1; *Arg2*, arginase 2; KO, knockout.

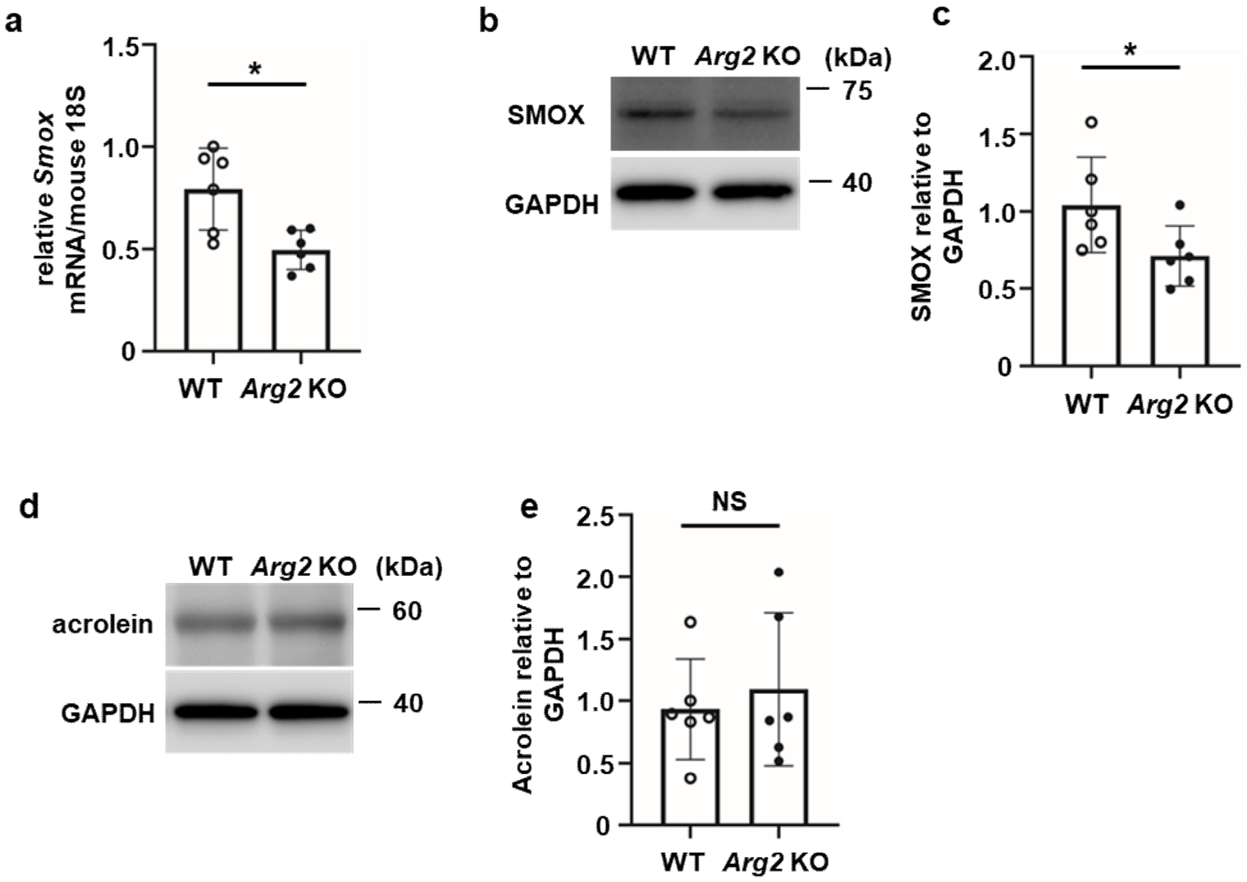

Supplementary Fig. S7. Spermine oxidase mRNA expression and protein levels are reduced in the *Arg2* KO UUO kidney, but acrolein is not altered.

(a) *Smox* mRNA expression determined by real-time PCR in WT and *Arg2* KO UUO kidneys (n = 6 in each group). Data are indicated as means ± SD. **(b)** SMOX protein levels in WT and *Arg2* KO UUO kidneys determined by western blotting. **(c)** Quantification of relative levels of SMOX normalized to GAPDH is shown (n = 6 in each group). Data are indicated as means ± SD. **(d)** Western blot analysis of acrolein in WT and *Arg2* KO UUO kidneys. **(e)** Quantification of relative levels of acrolein normalized to GAPDH is shown

(n = 6 in each group). Data are indicated as means ± SD. *$p < 0.05$. UUO, unilateral ureteral obstruction; SMOX, spermine oxidase; *Arg2*, arginase 2.

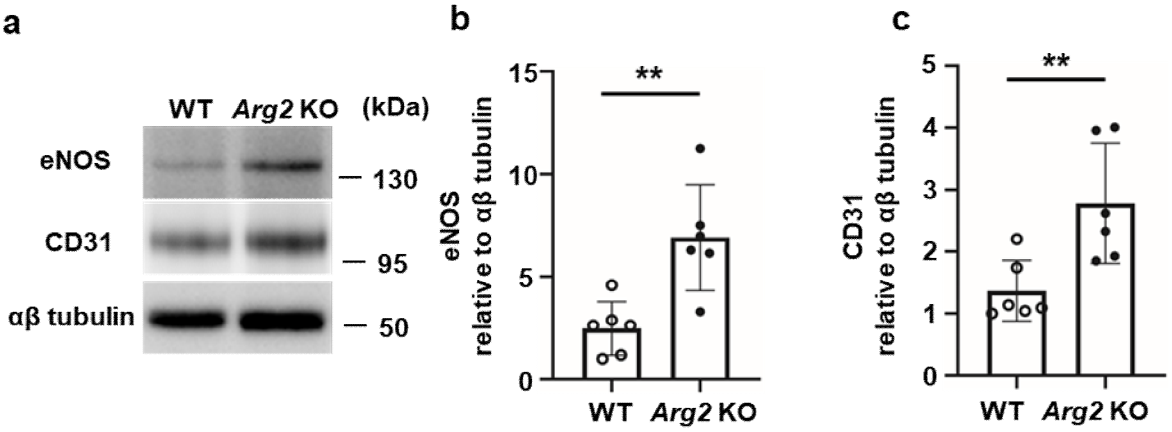

Supplementary Fig. S8. Protein levels of angiogenesis markers are increased in the *Arg2* KO UUO kidney.

(a) Western blot analysis of eNOS and CD31 protein in the UUO kidney of WT and *Arg2* KO mice. **(b)** eNOS and **(c)** CD31 relative protein levels normalized to αβ tubulin are shown (n = 6 in each group). Data are indicated as means ± SD. **p < 0.01. UUO, unilateral ureteral obstruction; eNOS, endothelial nitric oxide synthase.

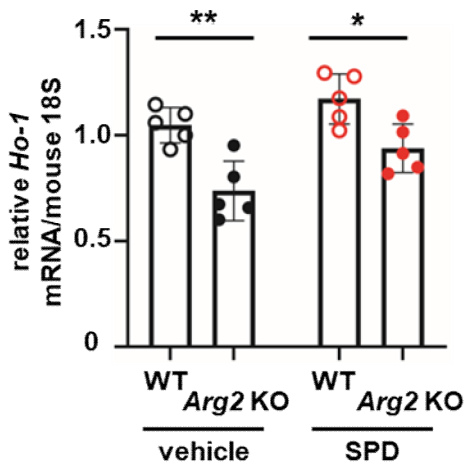

Supplementary Fig. S9. *HO-1* mRNA expression, which is reduced in Arg2 KO mice, tends to be restored with Spd treatment.

HO-1 mRNA expression determined by real-time PCR in Spd-treated WT and *Arg2* KO UUO kidneys (n = 5 in each group). Data are indicated as means ± SD. UUO, unilateral ureteral obstruction; Spd, spermidine.

Supplementary Table S1. Serum biochemistry and physiological parameters

	WT (n = 6)	*Arg2* KO (n = 6)
Body weight <g>	27.5 ± 3.0	26.9 ± 2.0
Systolic BP (before UUO) <mmHg>	107 ± 10	109 ± 7
Systolic BP (after UUO) <mmHg>	116 ± 9	126 ± 14
BUN <mg/dL>	42 ± 5	41 ± 7
Serum creatinine <mg/dL>	0.66 ± 0.38	0.58 ± 0.33

Data are expressed as the mean ± standard deviation or number.

WT, wild type; *Arg2* KO, arginase 2 knockout; BP, blood pressure; BUN, blood urea nitrogen; UUO, unilateral ureteral obstruction.

Supplementary Table S2. Primer sequences used for real-time PCR

Gene (*human*)	Forward primer sequence	Reverse primer sequence
ribosomal protein 18s	5′-AAACGGCTACCACATCCAAG-3′	5′-CCTCCAATGGATCCTCGTTA-3
collagen 1	5′-AGGGCTCCAACGAGATCGAGA-3′	5′-TACAGGAAGCAGACAGGGCCA-3′
endothelin-1	5′-CATCATTTGGGTCAACACTCC-3′	5′-CTTCCTCTCACTAACTGCTG-3′
GCLM	5′-CACAGCGAGGAGGAGTTTCC-3′	5′-ATCCAGCTGTGCAACTCCAA-3′
heme oxygenase-1	5′-TTGCCAGTGCCACCAAGTTC-3′	5′-TCAGCAGTCCCTGCAACTCC-3′
NQO1	5′-GACATCACAGGTAAACTGAAGG-3′	5′-GCAGGGGGAACTGGAATATC-3′

Gene (m*ouse*)	Forward primer sequence	Reverse primer sequence
ribosomal protein 18s	5′-AAGTTTCAGCACATCCTGCGAGTA-3′	5′-CCTCCAATGGATCCTCGTTA-3
heme oxygenase-1	5′-GCCGAGAATGCTGAGTTCAT-3′	5′-AGGAAGCCATCACCAGCTTA-3′
spermine oxidase	5′-AGCTGTACCCTCACCTACCC-3′	5′-TGTGGAGCTTCTATGCGCTGT-3′

PCR, polymerase chain reaction; GCLM, glutamate-cysteine ligase modifier subunit; NQO1, nicotinamide quinone oxidoreductase 1.

Supplementary Fig. S10. Uncropped western blots. The area surrounded by the red rectangle is shown in figures.

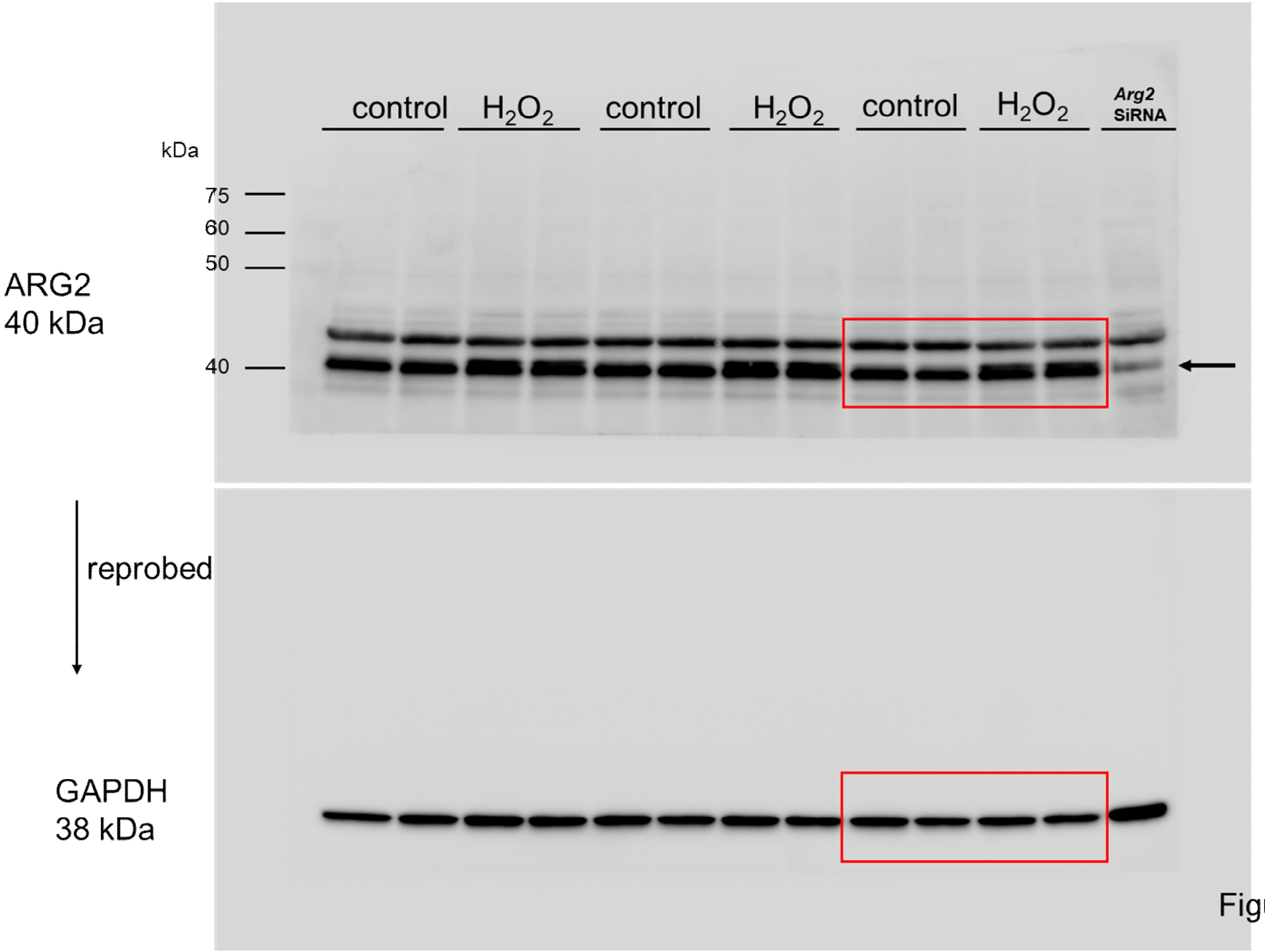

Figure 3a

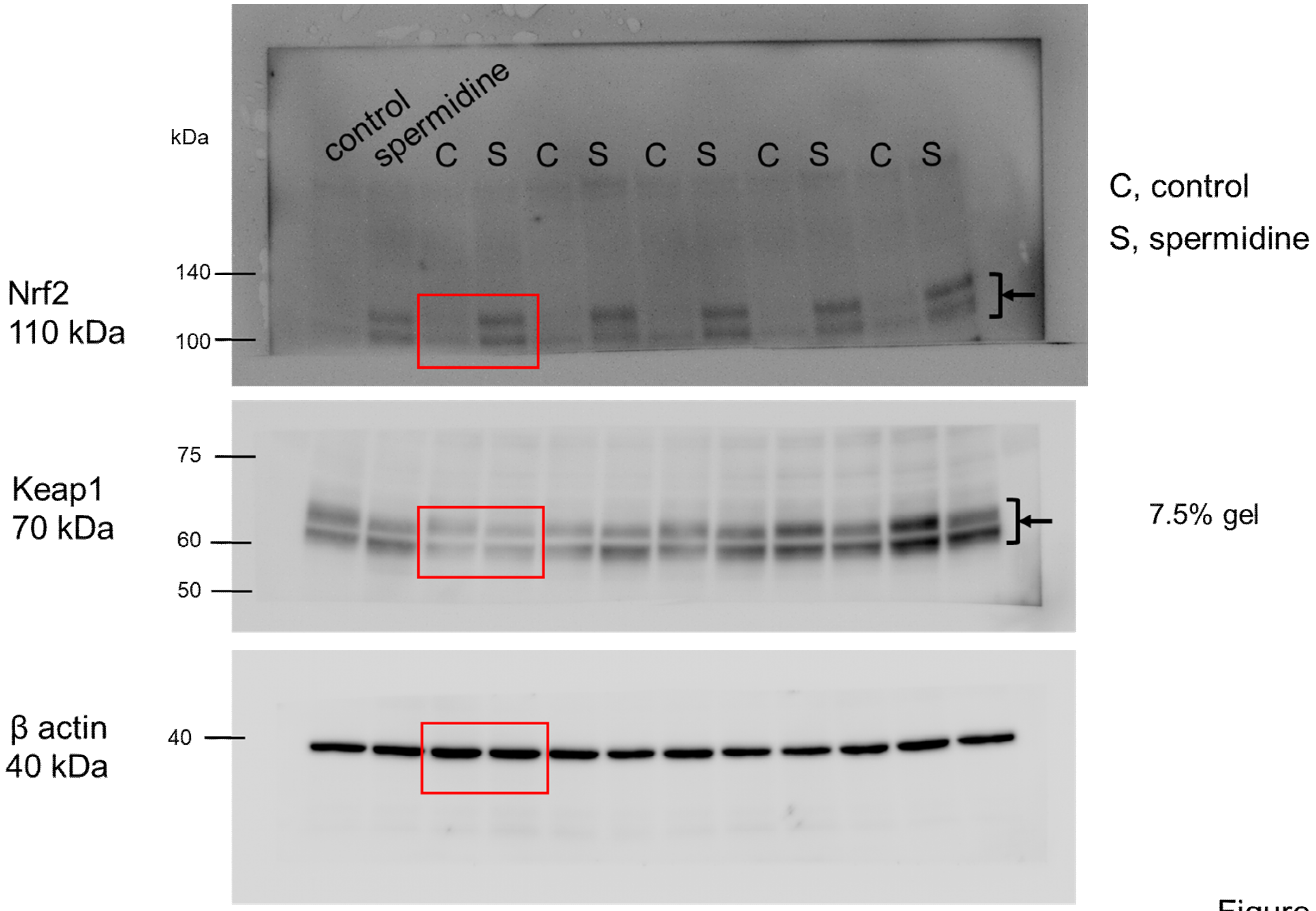

Figure 4a

165

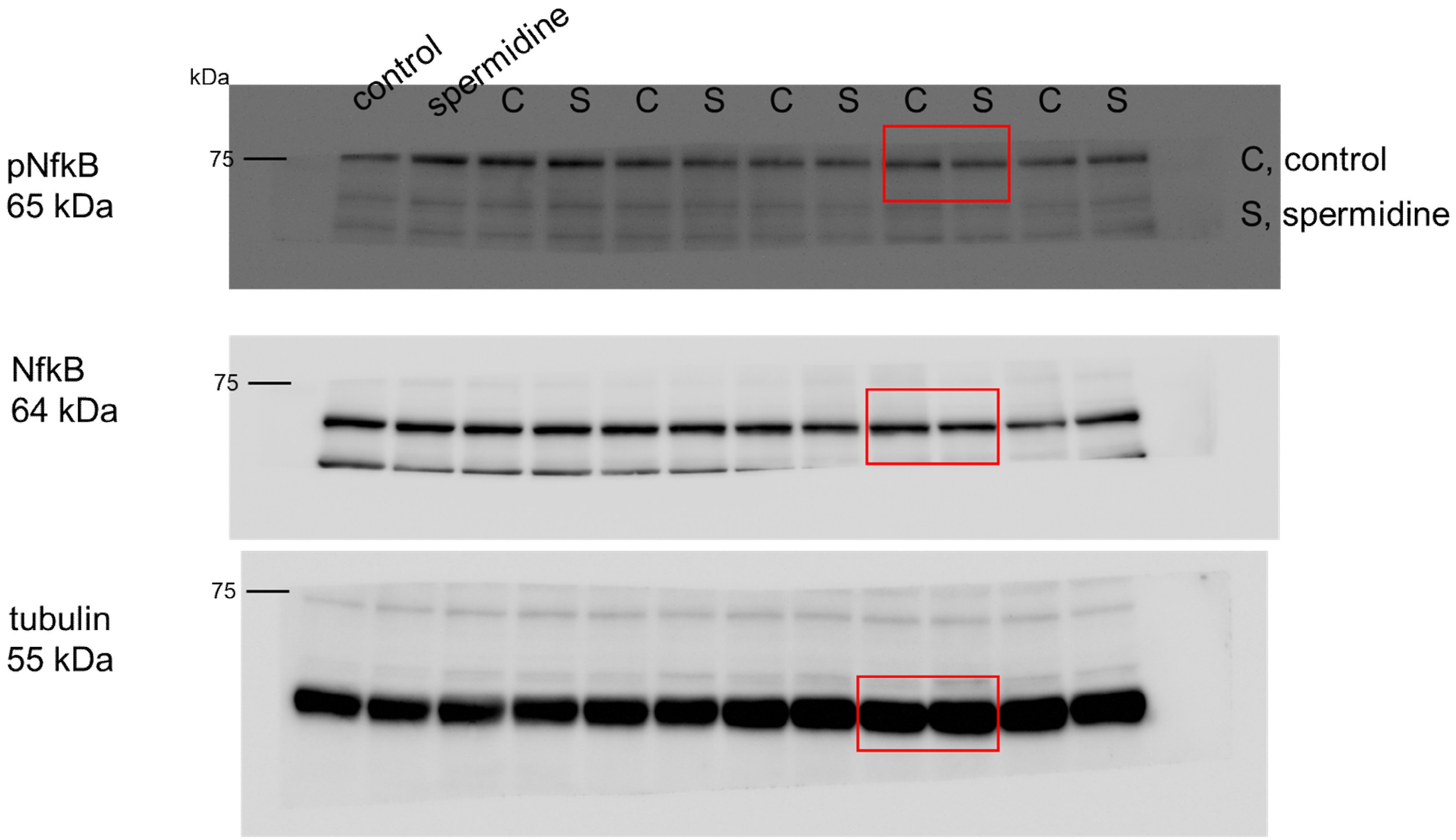

Figure 4d

166

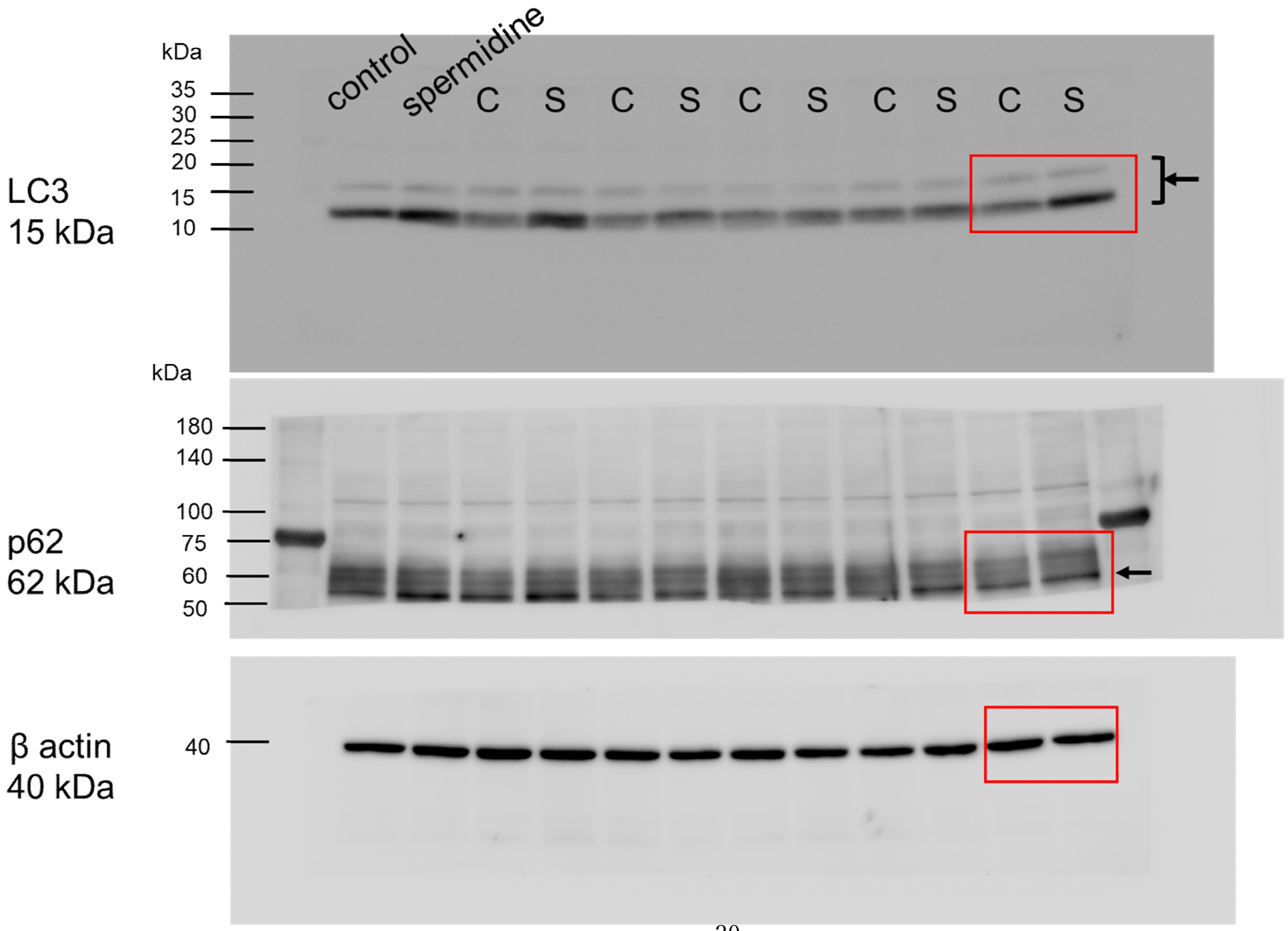

Figure 5a

167

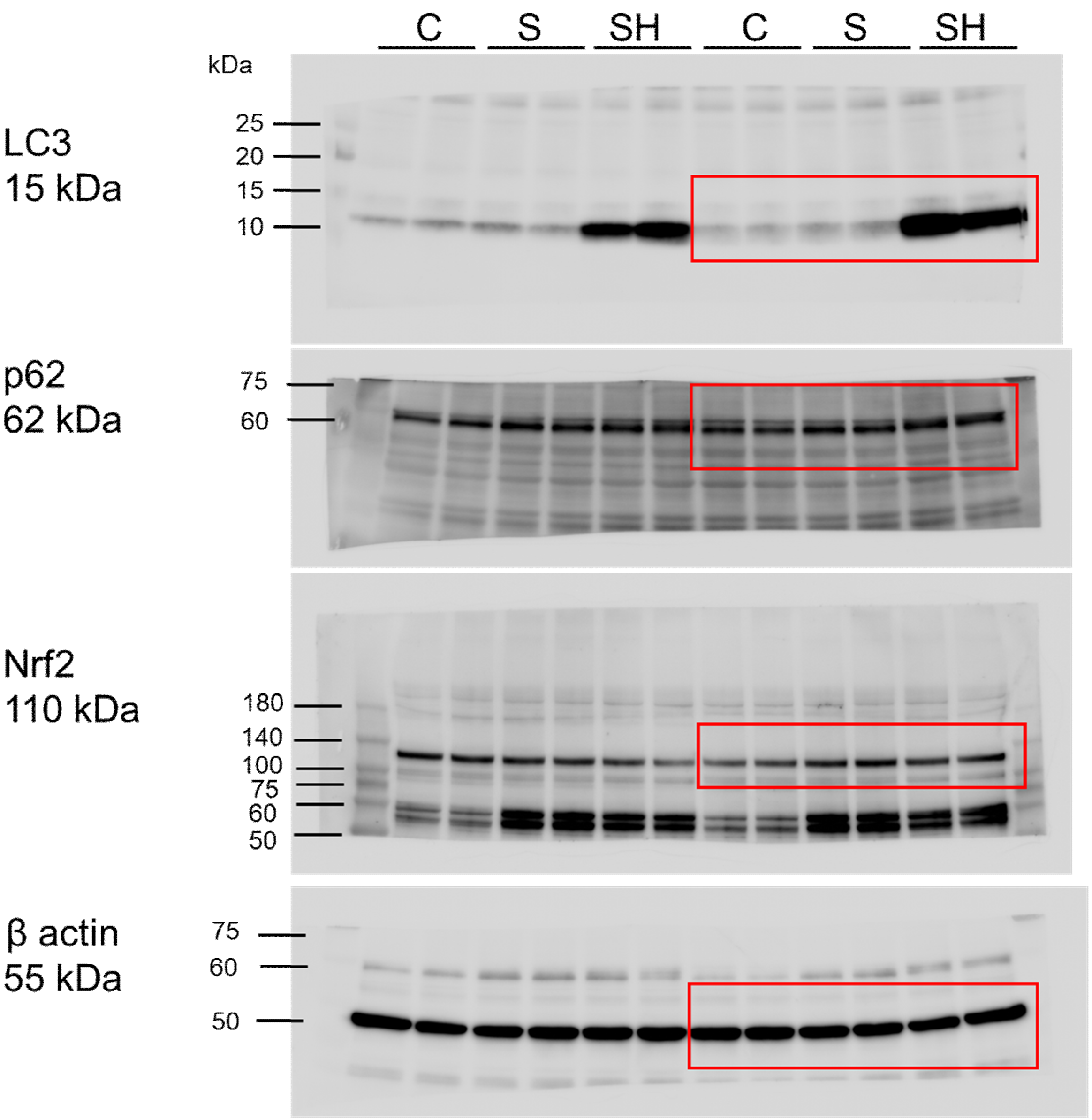

C, control
S, spermidine
SH, Spermidine + HCQ

Figure 5f

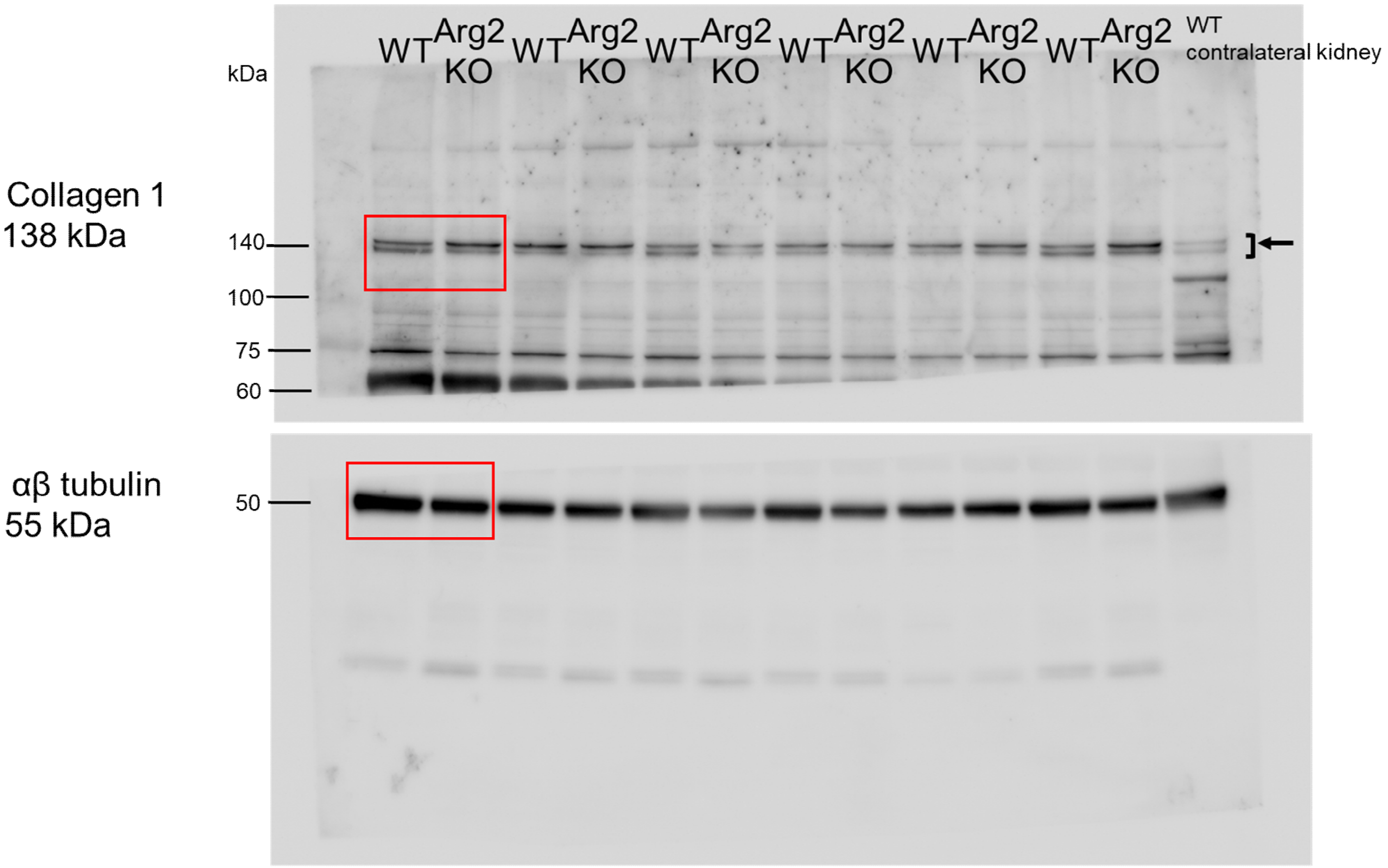

Figure 7g

169

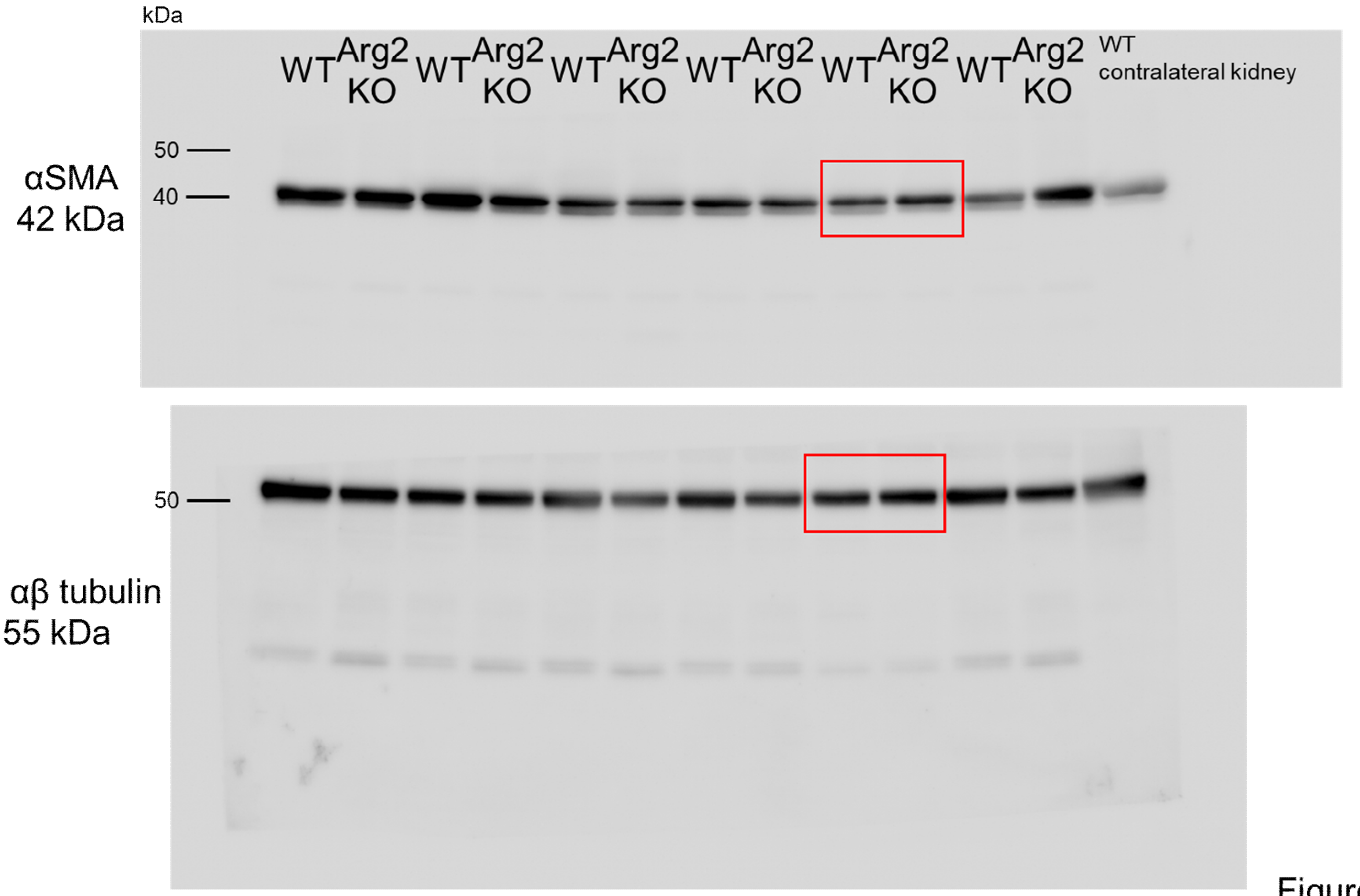

Figure 7h

170

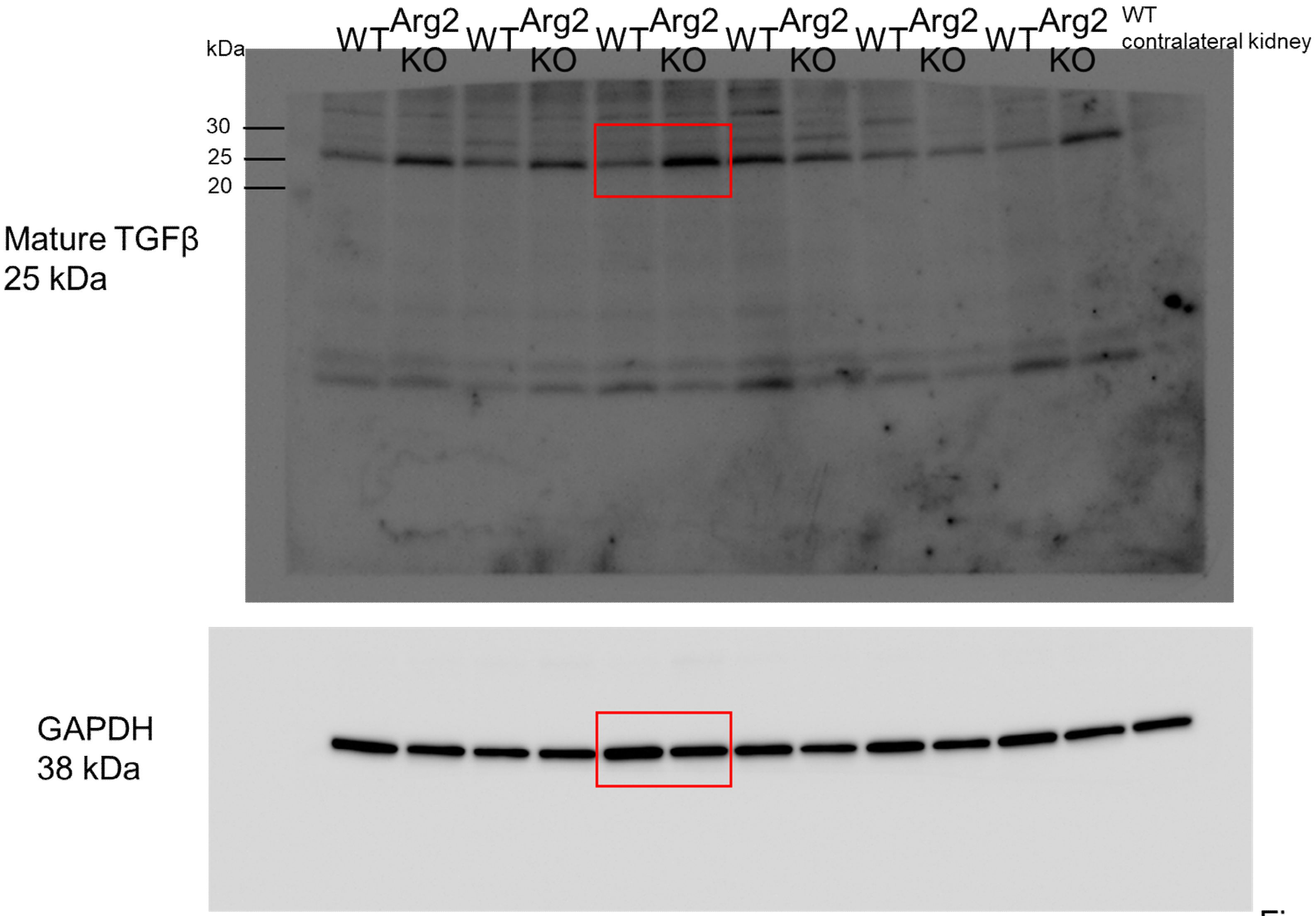

Figure 7i

171

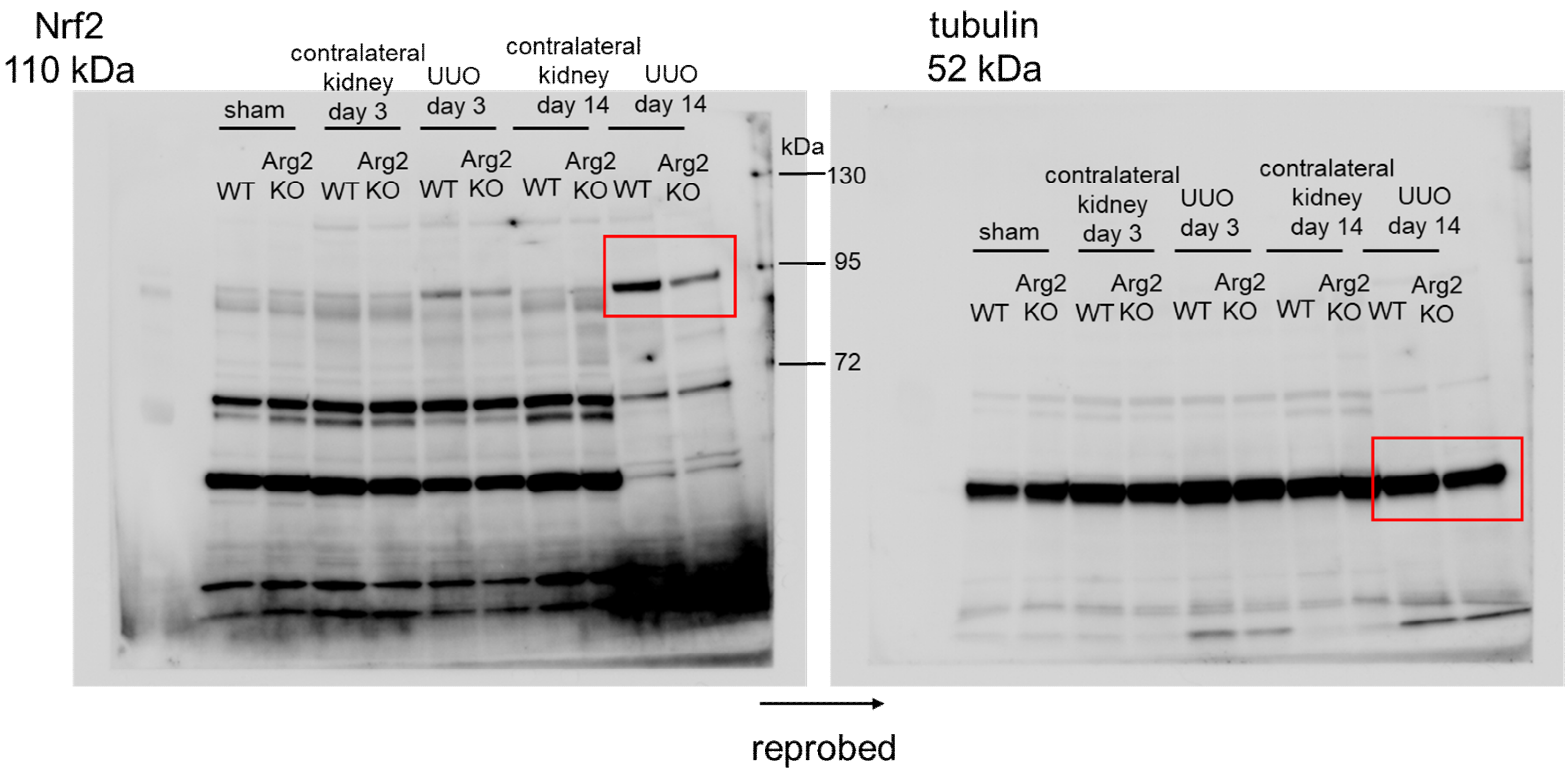

Figure 7j

172

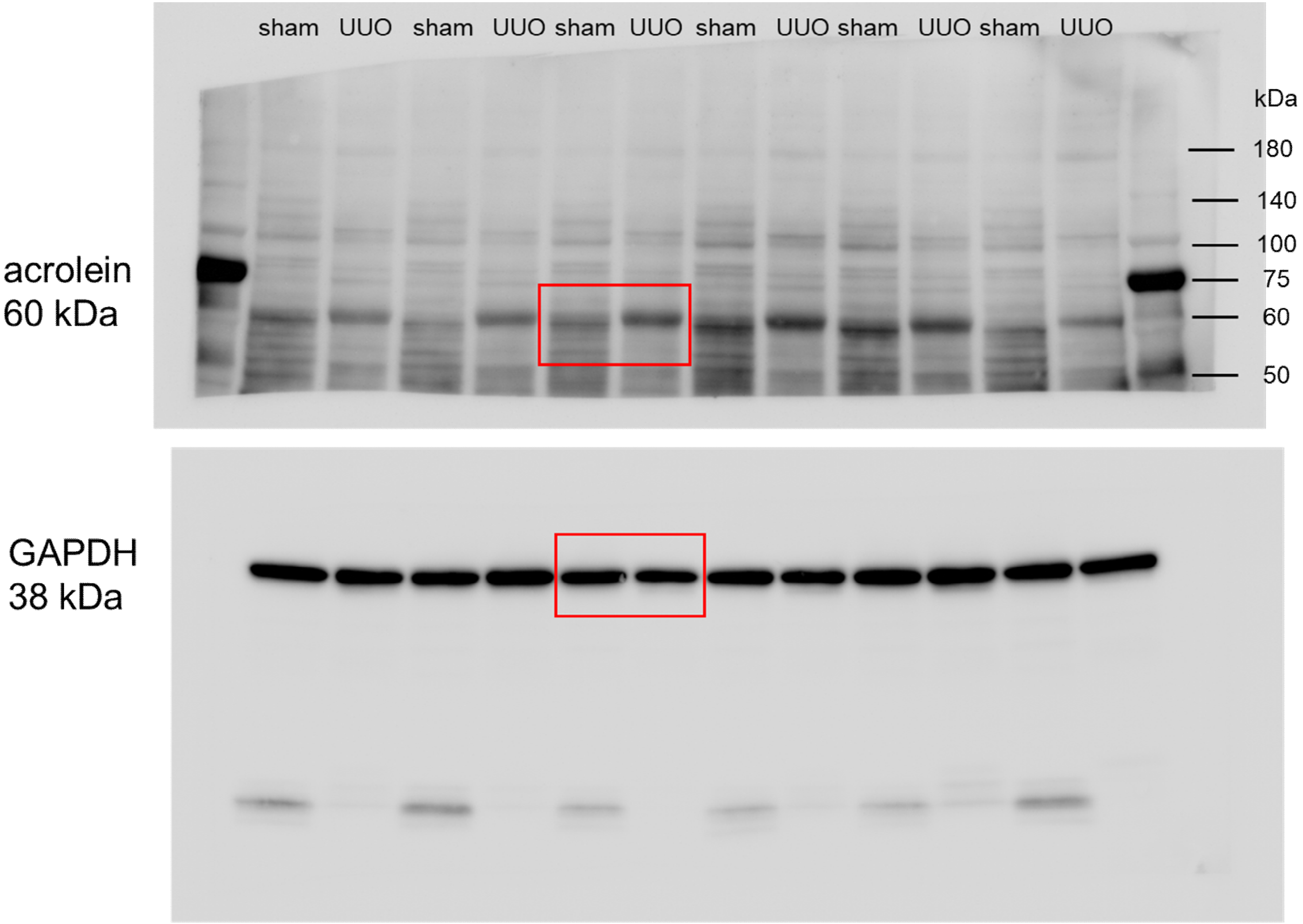

Figure S1a

173

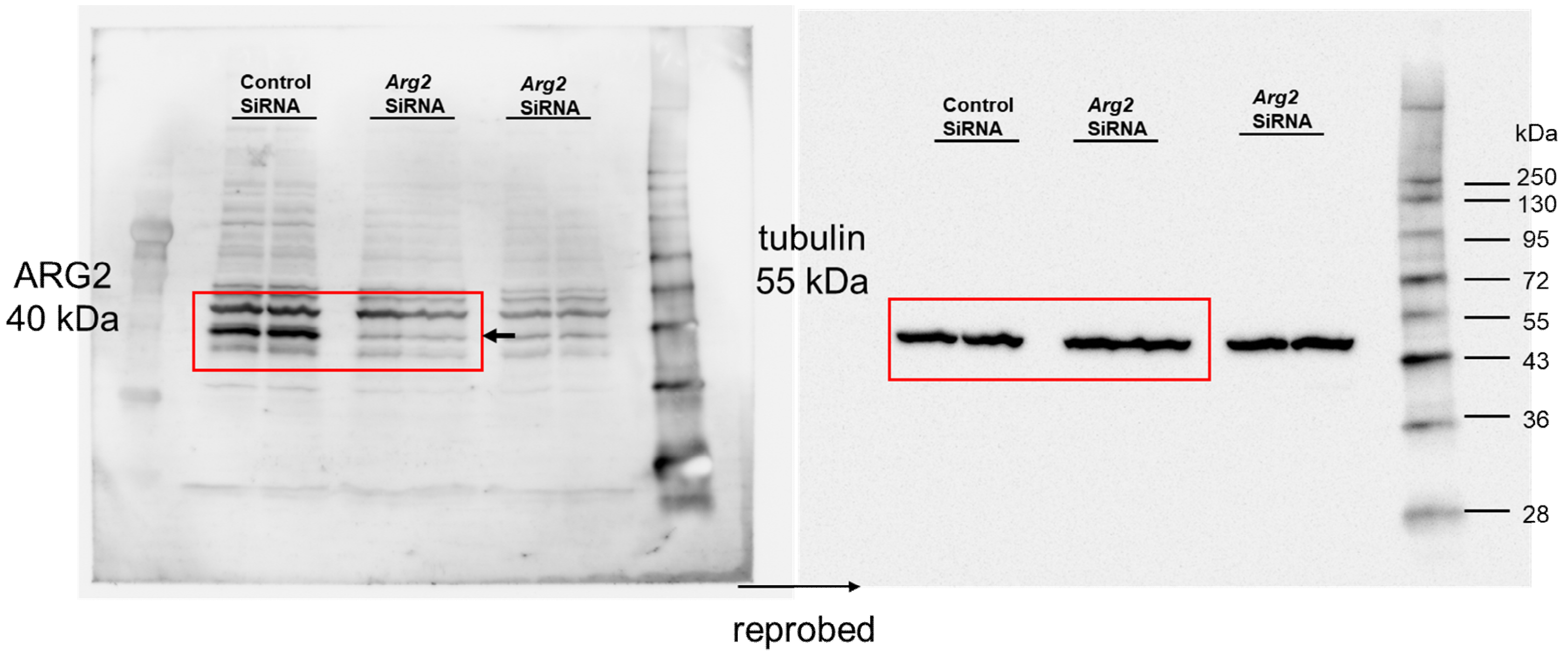

Figure S2a

174

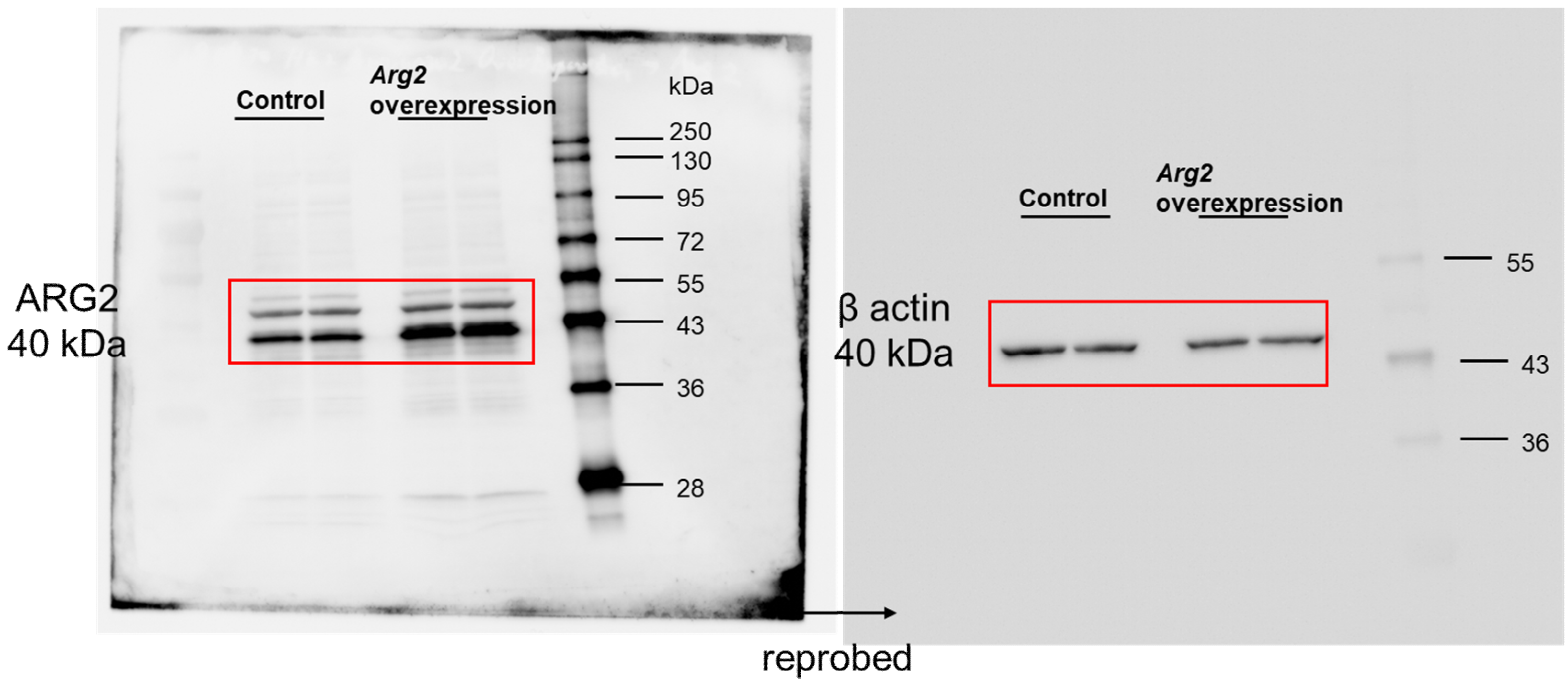

Control
Arg2 overexpression
kDa
250
130
95
72
55
43
36
28
ARG2
40 kDa
β actin
40 kDa
reprobed
Control
Arg2 overexpression
55
43
36

Figure S2b

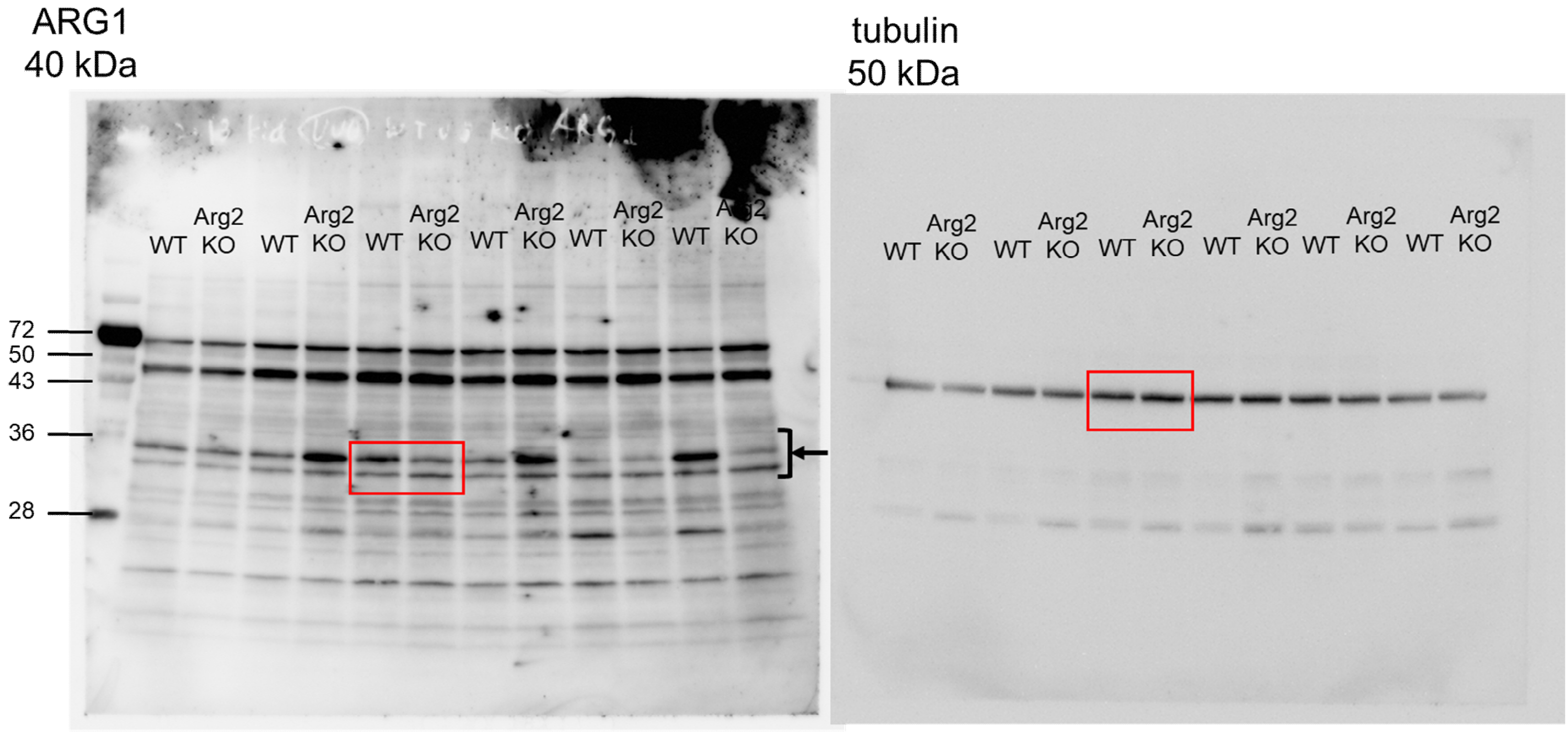

Figure S6a

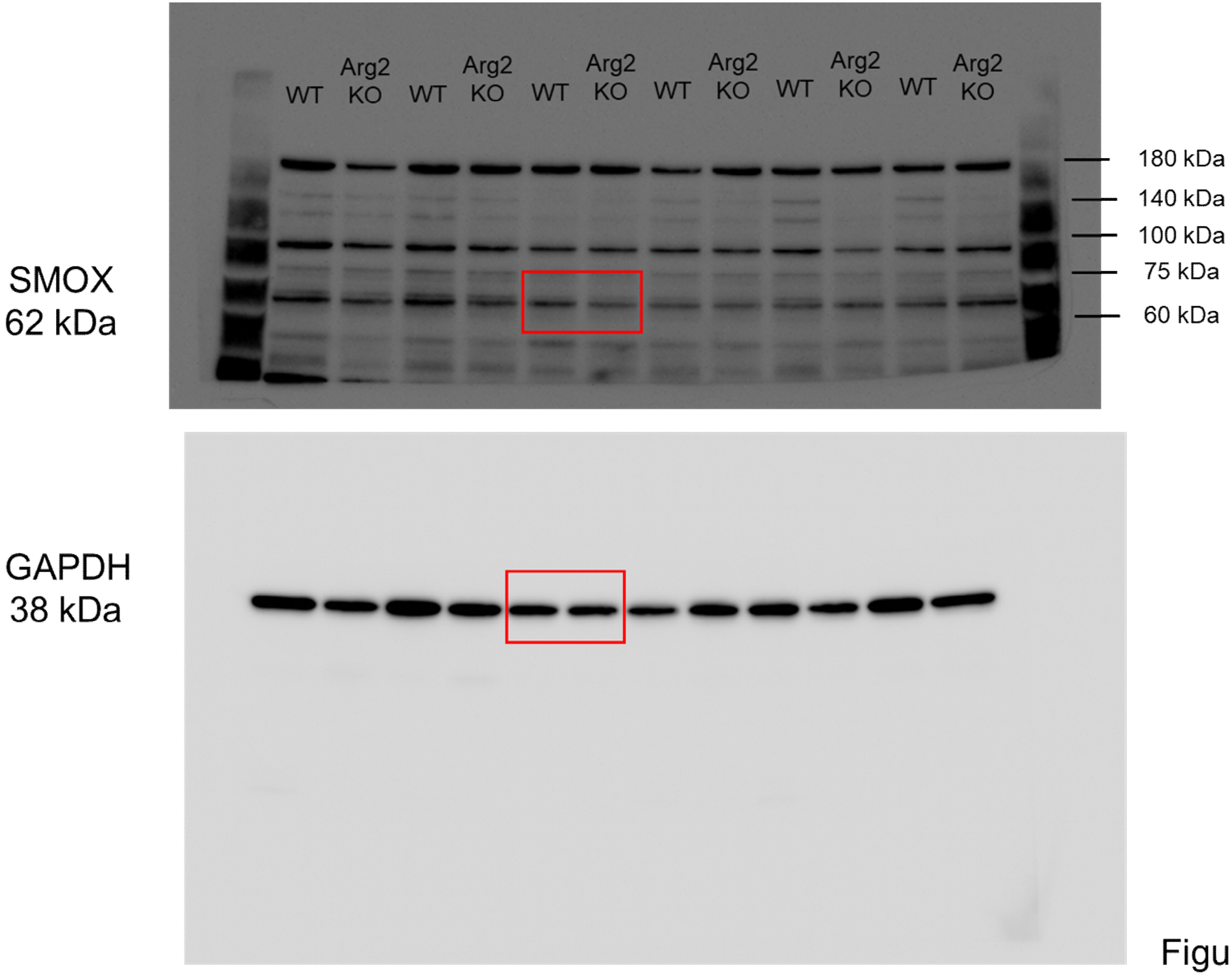
SMOX
62 kDa
WT
Arg2 KO
WT
Arg2 KO
WT
Arg2 KO
WT
Arg2 KO
WT
Arg2 KO
WT
Arg2 KO
180 kDa
140 kDa
100 kDa
75 kDa
60 kDa
GAPDH
38 kDa

Figure S7b

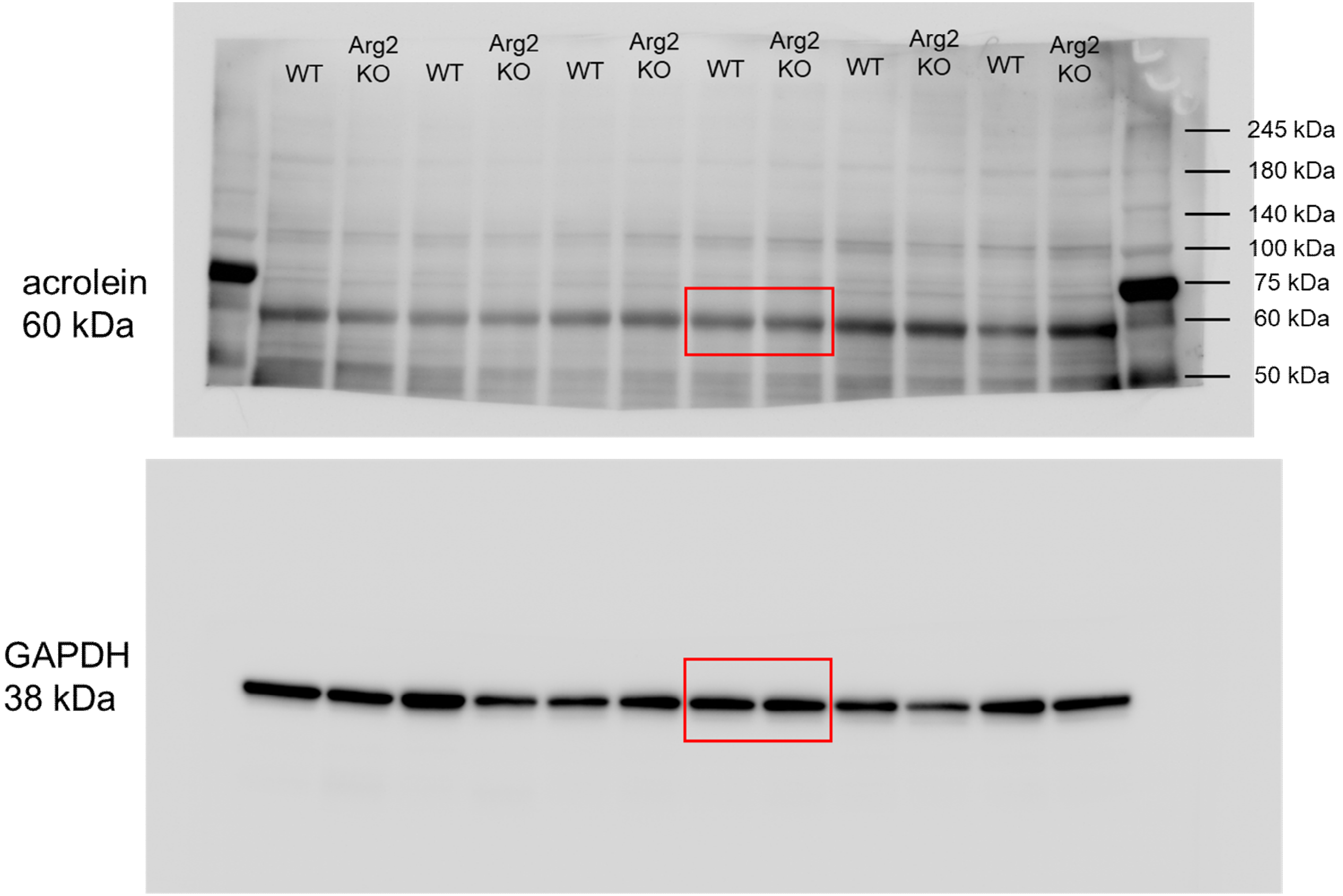

Figure S7d

179

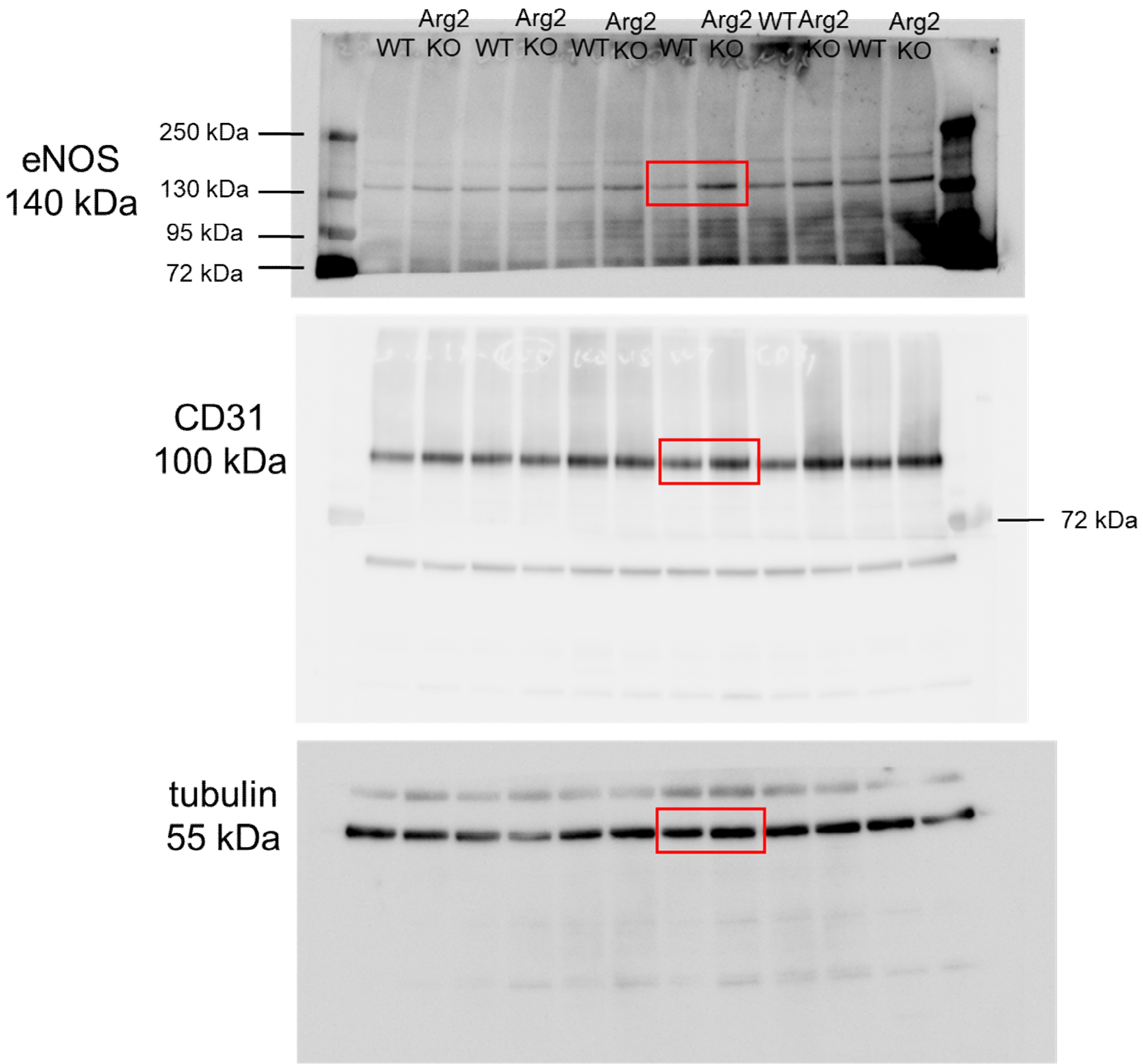
Arg2 Arg2 Arg2 Arg2 WT Arg2 Arg2
WT KO WT KO WT KO WT KO KO WT KO
eNOS
140 kDa
250 kDa
130 kDa
95 kDa
72 kDa
CD31
100 kDa
72 kDa
tubulin
55 kDa

Figure S8a

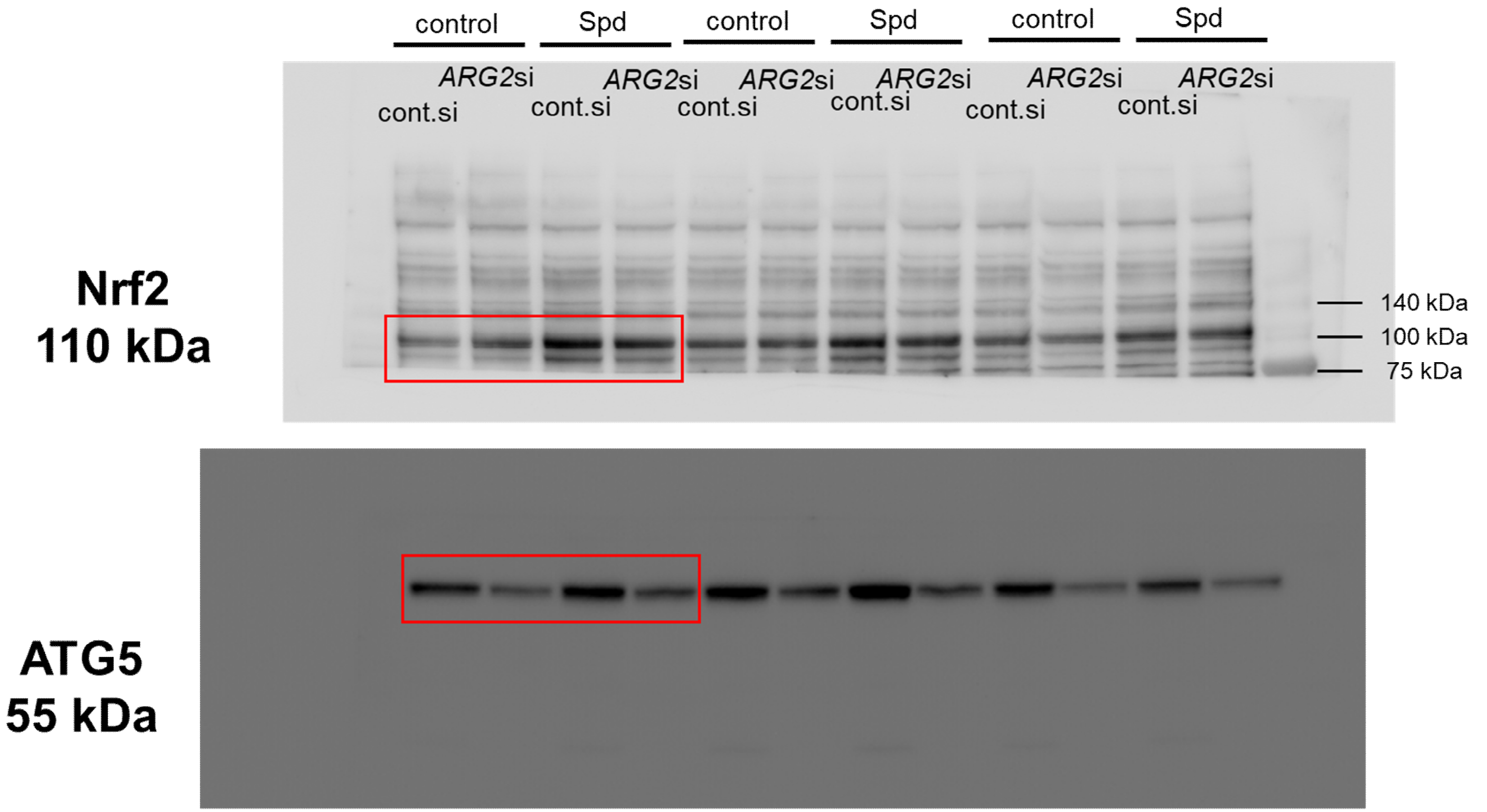

Figure S4b

181

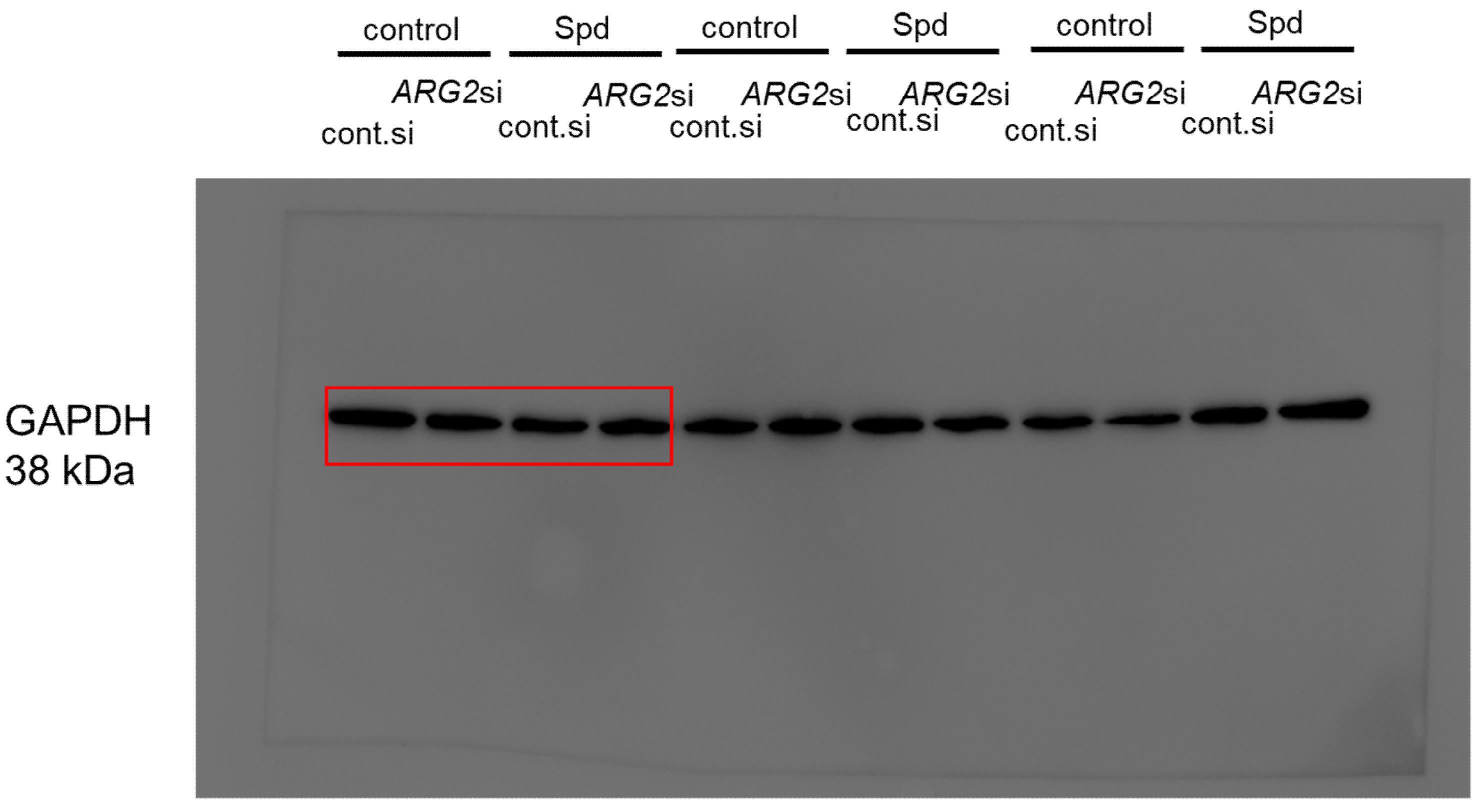

Figure S4b

182

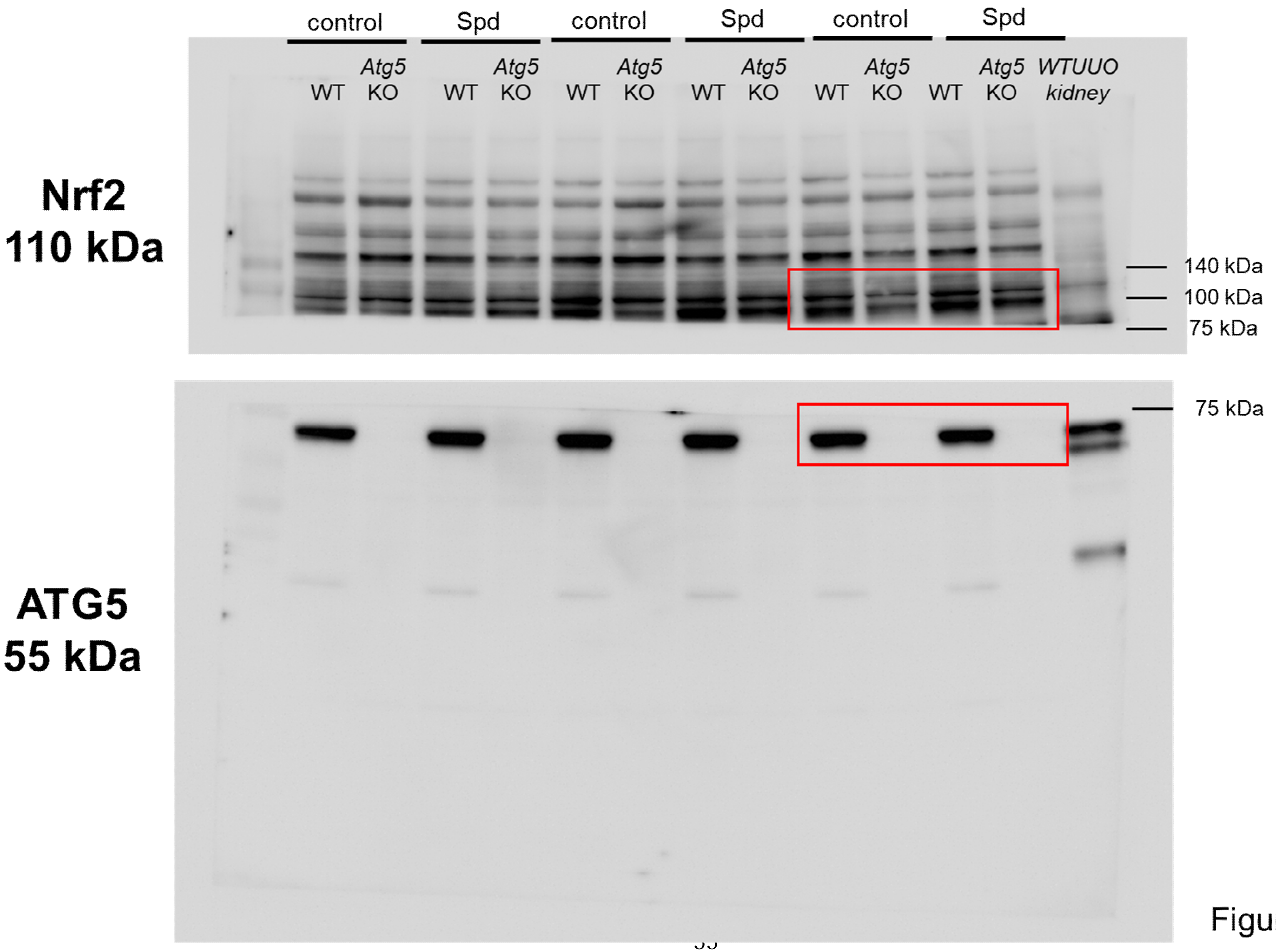

Nrf2
110 kDa
control
Spd
control
Spd
control
Spd
WT
Atg5 KO
WT
Atg5 KO
WT
Atg5 KO
WT
Atg5 KO
WT
Atg5 KO
WT
Atg5 KO
WTUUO kidney
140 kDa
100 kDa
75 kDa
ATG5
55 kDa
75 kDa

Figure S4e

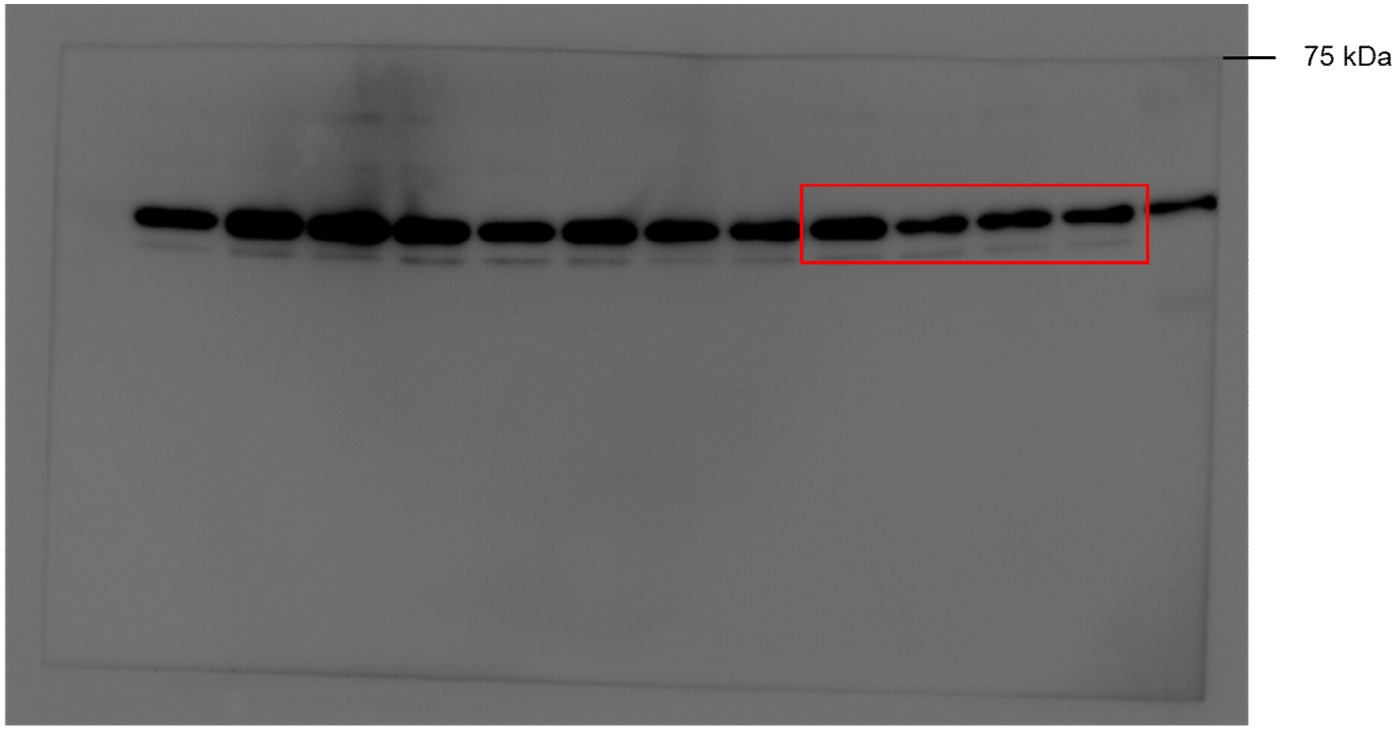

Figure S4e

184

185
