# Supplementary material for: Spermidine from arginine metabolism activates Nrf2 and inhibits kidney fibrosis
Source: Commun Biol. 2023 Jun 28;6:676. doi: 10.1038/s42003-023-05057-w (PMC10307812; doi:10.1038/s42003-023-05057-w)
Supplement: Supplementary file 3 — Reporting Summary [file 42003_2023_5057_MOESM3_ESM.pdf]

nature portfolio

Corresponding author(s): Kumiko Torisu, Toshiaki Nakano

Last updated by author(s): Jun 13, 2023

Reporting Summary

Nature Portfolio wishes to improve the reproducibility of the work that we publish. This form provides structure for consistency and transparency in reporting. For further information on Nature Portfolio policies, see our Editorial Policies and the Editorial Policy Checklist.

Statistics

For all statistical analyses, confirm that the following items are present in the figure legend, table legend, main text, or Methods section.

n/a	Confirmed	
☐	☒	The exact sample size (n) for each experimental group/condition, given as a discrete number and unit of measurement
☐	☒	A statement on whether measurements were taken from distinct samples or whether the same sample was measured repeatedly
☐	☒	The statistical test(s) used AND whether they are one- or two-sided<br>*Only common tests should be described solely by name; describe more complex techniques in the Methods section.*
☒	☐	A description of all covariates tested
☐	☒	A description of any assumptions or corrections, such as tests of normality and adjustment for multiple comparisons
☐	☒	A full description of the statistical parameters including central tendency (e.g. means) or other basic estimates (e.g. regression coefficient) AND variation (e.g. standard deviation) or associated estimates of uncertainty (e.g. confidence intervals)
☐	☒	For null hypothesis testing, the test statistic (e.g. F, t, r) with confidence intervals, effect sizes, degrees of freedom and P value noted<br>*Give P values as exact values whenever suitable.*
☒	☐	For Bayesian analysis, information on the choice of priors and Markov chain Monte Carlo settings
☒	☐	For hierarchical and complex designs, identification of the appropriate level for tests and full reporting of outcomes
☒	☐	Estimates of effect sizes (e.g. Cohen's d, Pearson's r), indicating how they were calculated

Our web collection on statistics for biologists contains articles on many of the points above.

Software and code

Policy information about availability of computer code

Data collection	N/A
Data analysis	N/A

For manuscripts utilizing custom algorithms or software that are central to the research but not yet described in published literature, software must be made available to editors and reviewers. We strongly encourage code deposition in a community repository (e.g. GitHub). See the Nature Portfolio guidelines for submitting code & software for further information.

Data

Policy information about availability of data

All manuscripts must include a data availability statement. This statement should provide the following information, where applicable:

- Accession codes, unique identifiers, or web links for publicly available datasets
- A description of any restrictions on data availability
- For clinical datasets or third party data, please ensure that the statement adheres to our policy

Data available on request from the authors.

Human research participants

Policy information about studies involving human research participants and Sex and Gender in Research.

Reporting on sex and gender	The sex of participants was determined based on self-report. The renal biopsy samples are equal in number of men and women in each group.
Population characteristics	Human kidney specimens were obtained in Kyushu University Hospital from donor kidneys at the time of living donor kidney transplantation (n = 6) or from a renal biopsy specimen diagnosed as IgA nephropathy (the score of tubular atrophy and interstitial fibrosis, T0, n = 6; T1, n =6; T2, n = 6). The age of the population is around 52 years and 50% are women. Further details can be found in Table 3.
Recruitment	Eligible patients were selected from all patients who were admitted to Kyushu University Hospital for renal biopsy for therapeutic purposes and whose consent was obtained.
Ethics oversight	Kyushu University Hospital

Note that full information on the approval of the study protocol must also be provided in the manuscript.

Field-specific reporting

Please select the one below that is the best fit for your research. If you are not sure, read the appropriate sections before making your selection.

☒ Life sciences ☐ Behavioural & social sciences ☐ Ecological, evolutionary & environmental sciences

For a reference copy of the document with all sections, see nature.com/documents/nr-reporting-summary-flat.pdf

Life sciences study design

All studies must disclose on these points even when the disclosure is negative.

Sample size	For mice experiment, there are 4-5 mice in each group. Sample size was determined according to previous reports. For human kidney samples, there are six in each group.
Data exclusions	No data were excluded from the analysis.
Replication	We confirmed that all attempts at replication were successful.
Randomization	All allocation was random.
Blinding	Renal histological analysis was performed by two investigators in a blinded fashion.

Reporting for specific materials, systems and methods

We require information from authors about some types of materials, experimental systems and methods used in many studies. Here, indicate whether each material, system or method listed is relevant to your study. If you are not sure if a list item applies to your research, read the appropriate section before selecting a response.

Materials & experimental systems

n/a	Involved in the study	
☐	☒	Antibodies
☐	☒	Eukaryotic cell lines
☒	☐	Palaeontology and archaeology
☐	☒	Animals and other organisms
☐	☒	Clinical data
☒	☐	Dual use research of concern

Methods

n/a	Involved in the study	
☒	☐	ChIP-seq
☒	☐	Flow cytometry
☒	☐	MRI-based neuroimaging

Antibodies

Antibodies used	rabbit ARG2 polyclonal antibody (bs11397-R, 1:100; Bioss), rabbit Spd polyclonal antibody (ab7318, 1:100; Abcam), rabbit anti-spermine oxidase (SMOX) antibody (15052-1-AP, 1:1000; Proteintech); rabbit anti-nuclear factor erythroid 2-related factor 2 (Nrf2)

	polyclonal antibody (ab137550, 1:1000; Abcam); rabbit anti-kelch-like ECH-associated protein 1 (Keap1) polyclonal antibody (ab139729, 1:1000; Abcam); anti-nuclear factor-κB (NFκB) polyclonal antibody (ab16502, 1:1000; Abcam); anti-phospho-NFκB polyclonal antibody (3033, 1:1000; Cell Signaling); mouse monoclonal anti-microtubule-associated protein 1A/1B-light chain 3 (LC3; CTB-LC3-2-IC, 1:1000; Cosmo Bio); anti-phospho-p62 antibody (95697S, 1:1000; Cell Signaling Technology); rabbit anti-collagen 1 antibody (ab34710, 1:5000; Abcam); mouse anti-α smooth muscle actin antibody (αSMA) (ab7817, 1:300; Abcam); rabbit TGFβ antibody (CST3711, 1:1000; Cell Signaling Technology); mouse monoclonal acrolein antibody (MA5-27553, 1:1000; Thermo Fischer Scientific); rabbit arginase 1 antibody (CST9819, 1:1000; Cell Signaling Technology); mouse anti-endothelial NOS/NOS type III (610297, 1:1000; BD Transduction Laboratories); anti-CD31 antibody (ab28364, 1:500; Abcam); rabbit anti-β-actin (1:5000, ab8227; Abcam); rabbit anti-αβ-tubulin antibody (2148, 1:5000; Cell Signaling Technology); and mouse anti-glyceraldehyde-3-phosphate dehydrogenase (GAPDH) antibody (ab8245, 1:5000; Abcam).
Validation	The specificity and applications of all primary antibodies were confirmed either on the manufacturer's website or in the data of the manuscript.

Eukaryotic cell lines

Policy information about cell lines and Sex and Gender in Research

Cell line source(s)	Human renal proximal tubule (HK-2) cells, male. Cell is derived from normal kidney. WT and Atg5 KO mouse embryonic fibroblasts were obtained from Riken Cell Bank (Tsukuba, Japan).
Authentication	None of the cells used were authenticated.
Mycoplasma contamination	This cell lines were tested negative for mycoplasma contamination by ATCC and RIKEN Cell Bank.
Commonly misidentified lines (See ICLAC register)	*Name any commonly misidentified cell lines used in the study and provide a rationale for their use.*

Animals and other research organisms

Policy information about studies involving animals; ARRIVE guidelines recommended for reporting animal research, and Sex and Gender in Research

Laboratory animals	Eight-week-old C57BL/6Jcl mice were obtained from CLEA Japan Inc. (Tokyo, Japan). Arg2 KO mice (Arg2tm1Weo/J) on a C57BL/6J background were purchased from Jackson Laboratory (Bar Harbor, ME, USA).
Wild animals	N/A
Reporting on sex	male
Field-collected samples	N/A
Ethics oversight	Kyushu University

Note that full information on the approval of the study protocol must also be provided in the manuscript.

Clinical data

Policy information about clinical studies
All manuscripts should comply with the ICMJE guidelines for publication of clinical research and a completed CONSORT checklist must be included with all submissions.

Clinical trial registration	N/A
Study protocol	*Note where the full trial protocol can be accessed OR if not available, explain why.*
Data collection	*Describe the settings and locales of data collection, noting the time periods of recruitment and data collection.*
Outcomes	*Describe how you pre-defined primary and secondary outcome measures and how you assessed these measures.*
